# Evaluating drug targets through human loss-of-function genetic variation

**DOI:** 10.1038/s41586-020-2267-z

**Published:** 2020-05-27

**Authors:** Eric Vallabh Minikel, Konrad J. Karczewski, Hilary C. Martin, Beryl B. Cummings, Nicola Whiffin, Daniel Rhodes, Jessica Alföldi, Richard C. Trembath, David A. van Heel, Mark J. Daly, Genome Aggregation Database Production Team, Jessica Alföldi, Irina M. Armean, Eric Banks, Louis Bergelson, Kristian Cibulskis, Ryan L. Collins, Kristen M. Connolly, Miguel Covarrubias, Beryl B. Cummings, Mark J. Daly, Stacey Donnelly, Yossi Farjoun, Steven Ferriera, Laurent Francioli, Stacey Gabriel, Laura D. Gauthier, Jeff Gentry, Namrata Gupta, Thibault Jeandet, Diane Kaplan, Konrad J. Karczewski, Kristen M. Laricchia, Christopher Llanwarne, Eric V. Minikel, Ruchi Munshi, Benjamin M. Neale, Sam Novod, Anne H. O’Donnell-Luria, Nikelle Petrillo, Timothy Poterba, David Roazen, Valentin Ruano-Rubio, Andrea Saltzman, Kaitlin E. Samocha, Molly Schleicher, Cotton Seed, Matthew Solomonson, Jose Soto, Grace Tiao, Kathleen Tibbetts, Charlotte Tolonen, Christopher Vittal, Gordon Wade, Arcturus Wang, Qingbo Wang, James S. Ware, Nicholas A. Watts, Ben Weisburd, Nicola Whiffin, Genome Aggregation Database Consortium, Carlos A. Aguilar Salinas, Tariq Ahmad, Christine M. Albert, Diego Ardissino, Gil Atzmon, John Barnard, Laurent Beaugerie, Emelia J. Benjamin, Michael Boehnke, Lori L. Bonnycastle, Erwin P. Bottinger, Donald W. Bowden, Matthew J. Bown, John C. Chambers, Juliana C. Chan, Daniel Chasman, Judy Cho, Mina K. Chung, Bruce Cohen, Adolfo Correa, Dana Dabelea, Mark J. Daly, Dawood Darbar, Ravindranath Duggirala, Josée Dupuis, Patrick T. Ellinor, Roberto Elosua, Jeanette Erdmann, Tõnu Esko, Martti Färkkilä, Jose Florez, Andre Franke, Gad Getz, Benjamin Glaser, Stephen J. Glatt, David Goldstein, Clicerio Gonzalez, Leif Groop, Christopher Haiman, Craig Hanis, Matthew Harms, Mikko Hiltunen, Matti M. Holi, Christina M. Hultman, Mikko Kallela, Jaakko Kaprio, Sekar Kathiresan, Bong-Jo Kim, Young Jin Kim, George Kirov, Jaspal Kooner, Seppo Koskinen, Harlan M. Krumholz, Subra Kugathasan, Soo Heon Kwak, Markku Laakso, Terho Lehtimäki, Ruth J. F. Loos, Steven A. Lubitz, Ronald C. W. Ma, Daniel G. MacArthur, Jaume Marrugat, Kari M. Mattila, Steven McCarroll, Mark I. McCarthy, Dermot McGovern, Ruth McPherson, James B. Meigs, Olle Melander, Andres Metspalu, Benjamin M. Neale, Peter M. Nilsson, Michael C. O’Donovan, Dost Ongur, Lorena Orozco, Michael J. Owen, Colin N. A. Palmer, Aarno Palotie, Kyong Soo Park, Carlos Pato, Ann E. Pulver, Nazneen Rahman, Anne M. Remes, John D. Rioux, Samuli Ripatti, Dan M. Roden, Danish Saleheen, Veikko Salomaa, Nilesh J. Samani, Jeremiah Scharf, Heribert Schunkert, Moore B. Shoemaker, Pamela Sklar, Hilkka Soininen, Harry Sokol, Tim Spector, Patrick F. Sullivan, Jaana Suvisaari, E. Shyong Tai, Yik Ying Teo, Tuomi Tiinamaija, Ming Tsuang, Teresa Dan Turner, Teresa Tusie-Luna, Erkki Vartiainen, Marquis P. Vawter, James. S. Ware, Hugh Watkins, Rinse K. Weersma, Maija Wessman, James G. Wilson, Ramnik J. Xavier, Stuart L. Schreiber, Daniel G. MacArthur

**Affiliations:** 1 Program in Medical and Population Genetics, Broad Institute of MIT and Harvard, Cambridge, MA USA; 2 Stanley Center for Psychiatric Research, Broad Institute of MIT and Harvard, Cambridge, MA USA; 3 Chemical Biology and Therapeutics Science Program, Broad Institute of MIT and Harvard, Cambridge, MA USA; 4 Analytical and Translational Genetics Unit, Massachusetts General Hospital, Boston, MA USA; 5 Program in Biological and Biomedical Sciences, Harvard Medical School, Boston, MA USA; 6 Henry and Allison McCance Center for Brain Health, Massachusetts General Hospital, Boston, MA USA; 7 Department of Neurology, Massachusetts General Hospital, Boston, MA USA; 8 Prion Alliance, Cambridge, MA USA; 9 Wellcome Sanger Institute, Hinxton, Cambridgeshire UK; 10 National Heart and Lung Institute and MRC London Institute of Medical Sciences, Imperial College London, London, UK; 11 Centre for Translational Bioinformatics, William Harvey Research Institute, Barts and the London School of Medicine and Dentistry, Queen Mary University of London and Barts Health NHS Trust, London, UK; 12 School of Basic and Medical Biosciences, Faculty of Life Sciences and Medicine, King’s College London, London, UK; 13 Blizard Institute, Barts and The London School of Medicine and Dentistry, Queen Mary University of London, London, UK; 14 Department of Chemistry & Chemical Biology, Harvard University, Cambridge, MA USA; 154 Present Address: Centre for Population Genomics, Garvan Institute of Medical Research and UNSW Sydney, Sydney, Australia; 155 Present Address: Centre for Population Genomics, Murdoch Children’s Research Institute, Melbourne, Australia; 15 European Molecular Biology Laboratory, European Bioinformatics Institute, Wellcome Genome Campus, Hinxton, Cambridge, UK; 16 Data Sciences Platform, Broad Institute of MIT and Harvard, Cambridge, MA USA; 17 Center for Genomic Medicine, Massachusetts General Hospital, Boston, MA USA; 18 Program in Bioinformatics and Integrative Genomics, Harvard Medical School, Boston, MA USA; 19 Genomics Platform, Broad Institute of MIT and Harvard, Cambridge, MA USA; 20 Broad Genomics, Broad Institute of MIT and Harvard, Cambridge, MA USA; 21 Division of Genetics and Genomics, Boston Children’s Hospital, Boston, MA USA; 22 Department of Pediatrics, Harvard Medical School, Boston, MA USA; 23 National Heart & Lung Institute and MRC London Institute of Medical Sciences, Imperial College London, London, UK; 24 Cardiovascular Research Centre, Royal Brompton & Harefield Hospitals NHS Trust, London, UK; 25 Unidad de Investigacion de Enfermedades Metabolicas, Instituto Nacional de Ciencias Medicas y Nutricion, Mexico City, Mexico; 26 Peninsula College of Medicine and Dentistry, Exeter, UK; 27 Division of Preventive Medicine, Brigham and Women’s Hospital and Harvard Medical School, Boston, MA USA; 28 Division of Cardiovascular Medicine, Brigham and Women’s Hospital and Harvard Medical School, Boston, MA USA; 29 Department of Cardiology, University Hospital, Parma, Italy; 30 Department of Biology, Faculty of Natural Sciences, University of Haifa, Haifa, Israel; 31 Department of Medicine, Albert Einstein College of Medicine, Bronx, NY USA; 32 Department of Genetics, Albert Einstein College of Medicine, Bronx, NY USA; 33 Department of Quantitative Health Sciences, Lerner Research Institute, Cleveland Clinic, Cleveland, OH USA; 34 Sorbonne Université, APHP, Gastroenterology Department, Saint Antoine Hospital, Paris, France; 35 Framingham Heart Study, National Heart, Lung, & Blood Institute and Boston University, Framingham, MA USA; 36 Department of Medicine, Boston University School of Medicine, Boston, MA USA; 37 Department of Epidemiology, Boston University School of Public Health, Boston, MA USA; 38 Department of Biostatistics and Center for Statistical Genetics, University of Michigan, Ann Arbor, MI USA; 39 National Human Genome Research Institute, National Institutes of Health, Bethesda, MD USA; 40 The Charles Bronfman Institute for Personalized Medicine, Icahn School of Medicine at Mount Sinai, New York, NY USA; 41 Department of Biochemistry, Wake Forest School of Medicine, Winston-Salem, NC USA; 42 Center for Genomics and Personalized Medicine Research, Wake Forest School of Medicine, Winston-Salem, NC USA; 43 Center for Diabetes Research, Wake Forest School of Medicine, Winston-Salem, NC USA; 44 Department of Cardiovascular Sciences and NIHR Leicester Biomedical Research Centre, University of Leicester, Leicester, UK; 45 Department of Epidemiology and Biostatistics, Imperial College London, London, UK; 46 Department of Cardiology, Ealing Hospital NHS Trust, Southall, UK; 47 Imperial College Healthcare NHS Trust, Imperial College London, London, UK; 48 Department of Medicine and Therapeutics, Chinese University of Hong Kong, Hong Kong, Hong Kong; 49 Program for Neuropsychiatric Research, McLean Hospital, Belmont, MA USA; 50 Department of Psychiatry, Harvard Medical School, Boston, MA USA; 51 Department of Medicine, University of Mississippi Medical Center, Jackson, MI USA; 52 Department of Epidemiology, Colorado School of Public Health, Aurora, CO USA; 53 Department of Medicine and Pharmacology, University of Illinois at Chicago, Chicago, IL USA; 54 Department of Genetics, Texas Biomedical Research Institute, San Antonio, TX USA; 55 Department of Biostatistics, Boston University School of Public Health, Boston, MA USA; 56 Framingham Heart Study, National Heart, Lung, & Blood Institute and Boston University, Framingham, MA USA; 57 Cardiac Arrhythmia Service and Cardiovascular Research Center, Massachusetts General Hospital, Boston, MA USA; 58 Cardiovascular Epidemiology and Genetics, Hospital del Mar Medical Research Institute (IMIM), Barcelona, Catalonia Spain; 59 Centro de Investigación Biomédica en Red Enfermedades Cardiovaculares (CIBERCV), Barcelona, Catalonia Spain; 60 Departament of Medicine, Medical School, University of Vic-Central University of Catalonia, Vic, Catalonia Spain; 61 Institute for Cardiogenetics, University of Lübeck, Lübeck, Germany; 62 DZHK (German Research Centre for Cardiovascular Research), partner site Hamburg/Lübeck/Kiel, Lübeck, Germany; 63 University Heart Center Lübeck, Lübeck, Germany; 64 Estonian Genome Center, Institute of Genomics, University of Tartu, Tartu, Estonia; 65 Clinic of Gastroenterology, Helsinki University and Helsinki University Hospital, Helsinki, Finland; 66 Institute of Clinical Molecular Biology (IKMB), Christian-Albrechts-University of Kiel, Kiel, Germany; 67 Cancer Genome Computational Analysis Group, Broad Institute of MIT and Harvard, Cambridge, MA USA; 68 Endocrinology and Metabolism Department, Hadassah-Hebrew University Medical Center, Jerusalem, Israel; 69 Department of Psychiatry and Behavioral Sciences, SUNY Upstate Medical University, Syracuse, NY USA; 70 Institute for Genomic Medicine, Columbia University Medical Center, Hammer Health Sciences, New York, NY USA; 71 Department of Genetics & Development, Columbia University Medical Center, Hammer Health Sciences, New York, NY USA; 72 Centro de Investigacion en Salud Poblacional, Instituto Nacional de Salud Publica, Cuernavaca, Mexico; 73 Genomics, Diabetes and Endocrinology, Lund University, Malmo, Sweden; 74 Lund University Diabetes Centre, Malmo, Sweden; 75 Human Genetics Center, University of Texas Health Science Center at Houston, Houston, TX USA; 76 Department of Neurology, Columbia University, New York, NY USA; 77 Institute of Genomic Medicine, Columbia University, New York, NY USA; 78 Institute of Biomedicine, University of Eastern Finland, Kuopio, Finland; 79 Department of Psychiatry, Helsinki University Central Hospital, Lapinlahdentie, Helsinki, Finland; 80 Department of Medical Epidemiology and Biostatistics, Karolinska Institutet, Stockholm, Sweden; 81 Department of Neurology, Helsinki University Central Hospital, Helsinki, Finland; 82 Institute for Molecular Medicine FIMM, University of Helsinki, Helsinki, Finland; 83 Department of Public Health, University of Helsinki, Helsinki, Finland; 84 Department of Medicine, Harvard Medical School, Boston, MA USA; 85 Center for Genome Science, Korea National Institute of Health, Chungcheongbuk-do, South Korea; 86 MRC Centre for Neuropsychiatric Genetics & Genomics, Cardiff University School of Medicine, Cardiff, UK; 87 Department of Health, THL-National Institute for Health and Welfare, Helsinki, Finland; 88 Section of Cardiovascular Medicine, Department of Internal Medicine, Yale School of Medicine, New Haven, CT USA; 89 Division of Pediatric Gastroenterology, Emory University School of Medicine, Atlanta, GA USA; 90 Department of Internal Medicine, Seoul National University Hospital, Seoul, South Korea; 91 Institute of Clinical Medicine, The University of Eastern Finland, Kuopio, Finland; 92 Kuopio University Hospital, Kuopio, Finland; 93 Department of Clinical Chemistry, Fimlab Laboratories and Finnish Cardiovascular Research Center-Tampere, Faculty of Medicine and Health Technology, Tampere University, Tampere, Finland; 94 The Mindich Child Health and Development Institute, Icahn School of Medicine at Mount Sinai, New York, NY USA; 95 Cardiac Arrhythmia Service, Massachusetts General Hospital, Boston, MA USA; 96 Department of Medicine and Therapeutics, The Chinese University of Hong Kong, Hong Kong, China; 97 Li Ka Shing Institute of Health Sciences, The Chinese University of Hong Kong, Hong Kong, China; 98 Hong Kong Institute of Diabetes and Obesity, The Chinese University of Hong Kong, Hong Kong, China; 99 Cardiovascular Research REGICOR Group, Hospital del Mar Medical Research Institute (IMIM), Barcelona, Catalonia Spain; 100 Department of Genetics, Harvard Medical School, Boston, MA USA; 101 Oxford Centre for Diabetes, Endocrinology and Metabolism, University of Oxford, Churchill Hospital, Headington, Oxford, UK; 102 Wellcome Centre for Human Genetics, University of Oxford, Oxford, UK; 103 Oxford NIHR Biomedical Research Centre, Oxford University Hospitals NHS Foundation Trust, John Radcliffe Hospital, Oxford, UK; 104 F Widjaja Foundation Inflammatory Bowel and Immunobiology Research Institute, Cedars-Sinai Medical Center, Los Angeles, CA USA; 105 Atherogenomics Laboratory, University of Ottawa Heart Institute, Ottawa, Canada; 106 Division of General Internal Medicine, Massachusetts General Hospital, Boston, MA USA; 107 Department of Clinical Sciences, University Hospital Malmo Clinical Research Center, Lund University, Malmo, Sweden; 108 Department of Clinical Sciences, Lund University, Skane University Hospital, Malmo, Sweden; 109 Instituto Nacional de Medicina Genómica (INMEGEN), Mexico City, Mexico; 110 Medical Research Institute, Ninewells Hospital and Medical School, University of Dundee, Dundee, UK; 111 Department of Molecular Medicine and Biopharmaceutical Sciences, Graduate School of Convergence Science and Technology, Seoul National University, Seoul, South Korea; 112 Department of Psychiatry, Keck School of Medicine at the University of Southern California, Los Angeles, CA USA; 113 Department of Psychiatry and Behavioral Sciences, Johns Hopkins University School of Medicine, Baltimore, MD USA; 114 Division of Genetics and Epidemiology, Institute of Cancer Research, London, UK; 115 Research Unit of Clinical Neuroscience, University of Oulu, Oulu, Finland; 116 Research Center, Montreal Heart Institute, Montreal, Quebec Canada; 117 Department of Medicine, Faculty of Medicine, Université de Montréal, Quebec, Canada; 118 Department of Public Health, Faculty of Medicine, University of Helsinki, Helsinki, Finland; 119 Department of Biomedical Informatics, Vanderbilt University Medical Center, Nashville, TN USA; 120 Department of Medicine, Vanderbilt University Medical Center, Nashville, TN USA; 121 Department of Biostatistics and Epidemiology, Perelman School of Medicine at the University of Pennsylvania, Philadelphia, PA USA; 122 Department of Medicine, Perelman School of Medicine at the University of Pennsylvania, Philadelphia, PA USA; 123 Center for Non-Communicable Diseases, Karachi, Pakistan; 124 National Institute for Health and Welfare, Helsinki, Finland; 125 NIHR Leicester Biomedical Research Centre, Glenfield Hospital, Leicester, UK; 126 Deutsches Herzzentrum München, Munich, Germany; 127 Technische Universität München, Munich, Germany; 128 Division of Cardiovascular Medicine, Nashville VA Medical Center and Vanderbilt University, School of Medicine, Nashville, TN USA; 129 Department of Psychiatry, Icahn School of Medicine at Mount Sinai, New York, NY USA; 130 Department of Genetics and Genomic Sciences, Icahn School of Medicine at Mount Sinai, New York, NY USA; 131 Institute for Genomics and Multiscale Biology, Icahn School of Medicine at Mount Sinai, New York, NY USA; 132 Institute of Clinical Medicine Neurology, University of Eastern Finland, Kuopio, Finland; 133 Department of Twin Research and Genetic Epidemiology, King’s College London, London, UK; 134 Department of Genetics and Psychiatry, University of North Carolina, Chapel Hill, NC USA; 135 Saw Swee Hock School of Public Health, National University of Singapore, National University Health System, Singapore, Singapore; 136 Department of Medicine, Yong Loo Lin School of Medicine, National University of Singapore, Singapore, Singapore; 137 Duke-NUS Graduate Medical School, Singapore, Singapore; 138 Life Sciences Institute, National University of Singapore, Singapore, Singapore; 139 Department of Statistics and Applied Probability, National University of Singapore, Singapore, Singapore; 140 Folkhälsan Institute of Genetics, Folkhälsan Research Center, Helsinki, Finland; 141 HUCH Abdominal Center, Helsinki University Hospital, Helsinki, Finland; 142 Center for Behavioral Genomics, Department of Psychiatry, University of California, San Diego, CA USA; 143 Institute of Genomic Medicine, University of California, San Diego, CA USA; 144 Juliet Keidan Institute of Pediatric Gastroenterology, Shaare Zedek Medical Center, The Hebrew University of Jerusalem, Jerusalem, Israel; 145 Instituto de Investigaciones Biomédicas UNAM, Mexico City, Mexico; 146 Instituto Nacional de Ciencias Médicas y Nutrición Salvador Zubirán, Mexico City, Mexico; 147 Department of Public Health Solutions, National Institute for Health and Welfare, Helsinki, Finland; 148 Radcliffe Department of Medicine, University of Oxford, Oxford, UK; 149 Department of Gastroenterology and Hepatology, University of Groningen and University Medical Center Groningen, Groningen, The Netherlands; 150 Department of Physiology and Biophysics, University of Mississippi Medical Center, Jackson, MS USA; 151 Program in Infectious Disease and Microbiome, Broad Institute of MIT and Harvard, Cambridge, MA USA; 152 Center for Computational and Integrative Biology, Massachusetts General Hospital, Boston, MA USA; 153 Department of Psychiatry & Human Behavior, University of California Irvine, Irvine, CA USA

**Keywords:** Target validation, Genomics

## Abstract

Naturally occurring human genetic variants that are predicted to inactivate protein-coding genes provide an in vivo model of human gene inactivation that complements knockout studies in cells and model organisms. Here we report three key findings regarding the assessment of candidate drug targets using human loss-of-function variants. First, even essential genes, in which loss-of-function variants are not tolerated, can be highly successful as targets of inhibitory drugs. Second, in most genes, loss-of-function variants are sufficiently rare that genotype-based ascertainment of homozygous or compound heterozygous ‘knockout’ humans will await sample sizes that are approximately 1,000 times those presently available, unless recruitment focuses on consanguineous individuals. Third, automated variant annotation and filtering are powerful, but manual curation remains crucial for removing artefacts, and is a prerequisite for recall-by-genotype efforts. Our results provide a roadmap for human knockout studies and should guide the interpretation of loss-of-function variants in drug development.

## Abstract (web summary)

Analysis of predicted loss-of-function variants from 125,748 human exomes and 15,708 whole genomes in the Genome Aggregation Database (gnomAD) provides a roadmap for human ‘knockout’ studies and a guide for future research into disease biology and drug-target selection.

## Main

Human genetics is an increasingly crucial source of evidence guiding the selection of new targets for drug discovery^1^. Most new clinical drug candidates eventually fail for lack of efficacy^2^, and although in vitro, cell culture and animal model systems can provide preclinical evidence that the compound engages its target, too often the target itself is not causally related to human disease^1^. Candidates targeting genes with human genetic evidence for disease causality are more likely to reach approval^3,4^, and identification of humans with loss-of-function (LoF) variants, particularly two-hit (homozygous or compound heterozygous) genotypes, has, for several genes, correctly predicted the safety and phenotypic effect of pharmacological inhibition^5^. Although these examples demonstrate the value of human genetics in drug development, important questions remain regarding strategies for identifying individuals with LoF variants in a gene of interest, interpretation of the frequency—or lack—of such individuals, and whether it is wise to pharmacologically target a gene in which LoF variants are associated with a deleterious phenotype.

Public databases of human genetic variation have catalogued predicted loss-of-function (pLoF) variants—nonsense, essential splice site, and frameshift variants expected to result in a non-functional allele. This presents an opportunity to study the effects of pLoF variation in genes of interest and to identify individuals with pLoF genotypes to understand gene function or disease biology, or to assess potential for therapeutic targeting. Although many variants initially annotated as pLoF do not, in fact, abolish gene function^6^, rigorous automated filtering can remove common error modes^7^. True LoF variants are generally rare, and show important differences between outbred, bottlenecked^8^ and consanguineous^9^ populations^6,10^. Counting the number of distinct pLoF variants in each gene in a population sample allows the quantification of gene essentiality in humans through a metric named ‘constraint’^10–13^. Specifically, the rate at which de novo pLoF mutations arise in each gene is predicted on the basis of rates of DNA mutation^10,12^, and the ratio of the count of pLoF variants observed in a database to the number expected based on mutation rates—obs/exp, or constraint score—measures how strongly purifying natural selection has removed such variants from the population. The annotation of pLoF variants remains imperfect, and continued improvements are being made^14^, but constraint usefully measures gene essentiality, as demonstrated by agreement with cell culture and mouse knockout experiments^7^, by overlap with human disease genes^7,10^ and genes depleted for structural variation^15^, and by the power of constraint to enrich for deleterious variants in neurodevelopmental disorders^7,16^.

Building on these insights, here we leverage pLoF variation in the Genome Aggregation Database (gnomAD)^7^ v2 dataset of 141,456 individuals to answer open questions in the interpretation of human pLoF variation in disease biology and drug development.

## Constraint in human drug targets

**Fig. 1:**
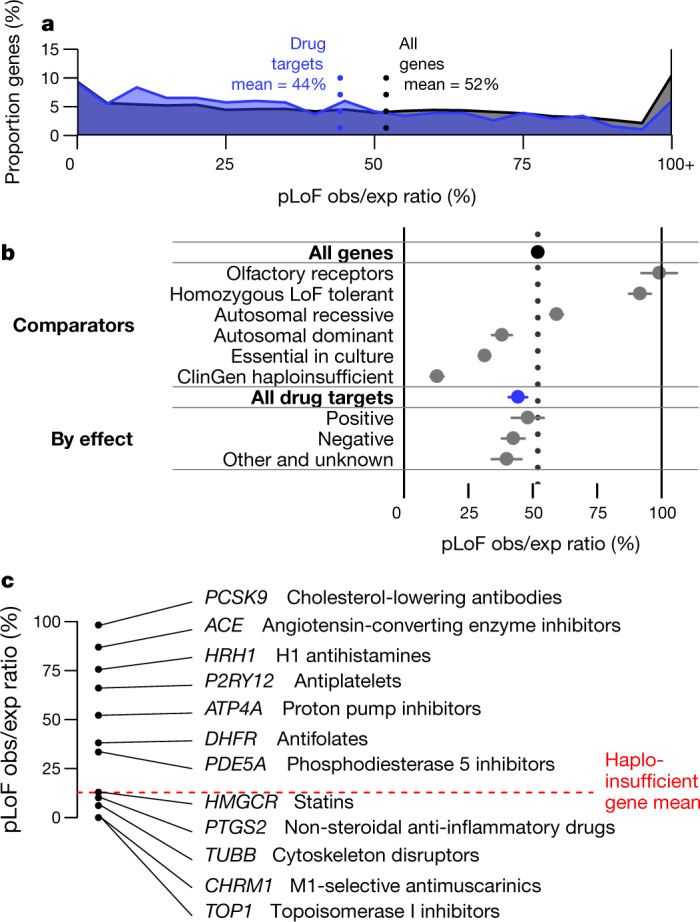
pLoF constraint in drug targets. **a**, Histogram of pLoF obs/exp values for all genes (black, *n* = 17,604) versus drug targets (blue, *n* = 383). **b**, Forest plot of means (dots) and 95% confidence intervals of the mean (line segments), for constraint in the indicated gene sets (data sources and *n* values in Extended Data Table 1). For drug effect, ‘positive’ indicates agonist, activator or inducer, whereas negative indicates antagonist, inhibitor or suppressor, for example. **c**, Examples of drug targets and corresponding drug classes from across the constraint spectrum. Details in Extended Data Table 2.

**Extended Data Fig. 1:**
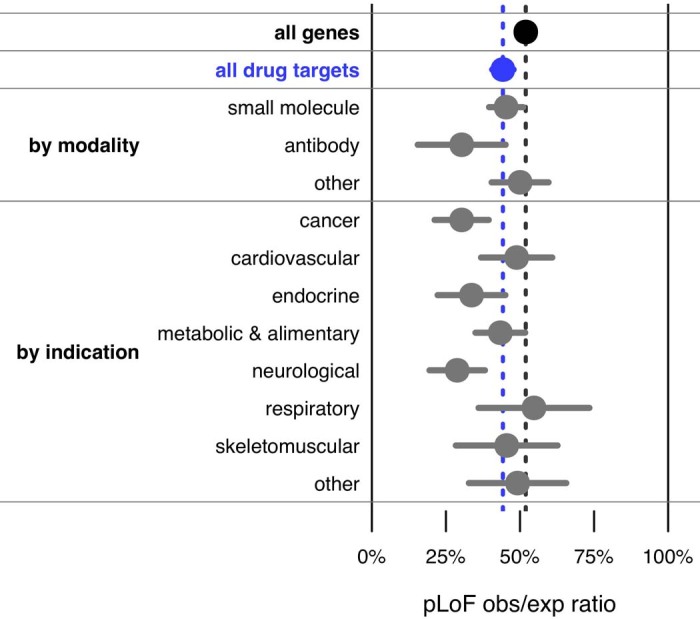
Drug target constraint by modality and indication. Mean (dots) and 95% confidence interval (line segments) for constraint in subsets of drug-targets sets (data sources and number of genes for each list are provided in Extended Data Table 1). Modality information was extracted from DrugBank and indication information from ATC codes; see Extended Data Table 1 for details.

We compared constraint in the targets of approved drugs extracted from DrugBank^17^ (*n* = 383) versus all protein-coding genes (*n =* 17,604). Drug targets were, on average, just slightly more constrained than all genes (mean 44% versus 52%, nominal *P* = 0.00028, *D* = 0.11, two-sided Kolmogorov–Smirnov test), but the two gene sets had a qualitatively similar distribution of scores, ranging from intensely constrained (0% obs/exp) to not at all constrained (≥100% obs/exp) (Fig. 1a). Constraint scores showed clear divergence between categories of genes (Extended Data Table 1) expected to be more or less tolerant of inactivation (Fig. 1b), as previously reported^7,10^, validating the usefulness of constraint as a measure of gene essentiality. Nonetheless, when drug targets were stratified by drug effect (Fig. 1b), modality, or indication (Extended Data Fig. 1), no statistically significant differences between subsets of drug targets were observed.

**Extended Data Fig. 2:**
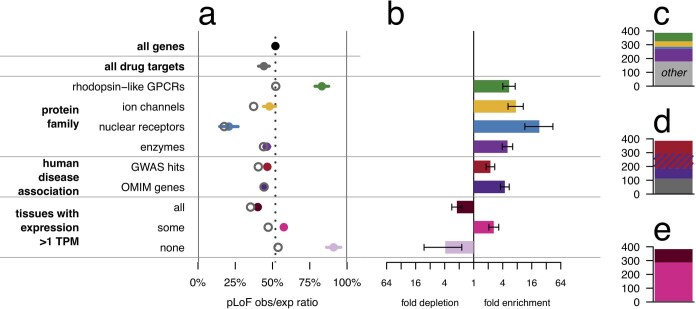
Drug-target gene set confounding. **a**, Forest plot of means (dots) and 95% confidence intervals of the mean (line segments) for gene sets evaluated for confounding with drug-target status. Data sources and number of genes for each list are provided in Extended Data Table 1. LoF obs/exp ratios differ significantly from the set of all genes for four canonically druggable protein families (top), human disease-associated genes (middle), and genes by broadness of tissue expression (bottom). Within each class, the genes that are drug targets have a lower mean obs/exp ratio (hollow grey circles) than the class overall**. b**, The druggable protein families, disease-associated genes, and genes expressed in some tissues but not others are enriched several-fold among the set of drug targets. Bars indicate fold enrichment and error bars indicate 95% confidence intervals. **c**–**e**, Composition of drug targets when broken down by protein family (**c**), disease association (**d**), or broadness of tissue expression (**e**). The enriched classes account for most drug targets. In a linear model, after controlling for protein family, disease association status, and number of tissues with expression >1 transcript per million (TPM), drug targets are still more constrained than other genes (−8.0% obs/exp, nominal *P* = 0.00011, *t* = −3.9, df = 17,325 for the contribution of drug_target in the linear regression obs/exp ~ drug_target + family + dz_assoc + n_tissues), but the probable existence of additional unobserved confounders cautions against over-interpretation of this observation (see main text).

The slightly but significantly lower obs/exp value among drug targets may superficially appear to provide evidence that constrained genes make superior drug targets. Stratification of drug targets by protein family, human disease association, and tissue expression, however, argues against this interpretation. Drug targets are strongly enriched for a few canonically ‘druggable’ protein families, for genes known to be involved in human disease, and for genes with tissue-restricted expression; each of these properties is in turn correlated with either significantly stronger or weaker constraint (Extended Data Fig. 2). Although controlling for these correlations does not abolish the trend of stronger constraint among drug targets, the correlation of so many observed variables with the status of a gene as a drug target argues that many unobserved variables probably also confound interpretation of the lower mean obs/exp value among drug targets.

The overall constraint distribution of drug targets (Fig. 1a) also argues against the view that a gene in which LoF is associated with a deleterious phenotype cannot be successfully targeted. Indeed, 19% of drug targets (*n* = 73), including 52 targets of inhibitors, antagonists or other ‘negative’ drugs, have lower obs/exp values than the average (12.8%) for genes known to cause severe diseases of haploinsufficiency^18^ (ClinGen level 3). To determine whether this finding could be explained by a particular class or subset of drugs, we examined constraint in several well-known example drug targets (Fig. 1c, Extended Data Table 2). Some heavily constrained genes are targets of cytotoxic chemotherapy agents such as topoisomerase inhibitors or cytoskeleton disruptors, a set of drugs intuitively expected to target essential genes. However, genes with near-complete selection against pLoF variants also include *HMGCR* and *PTGS2*, the targets of highly successful, chronically used inhibitors—statins and aspirin.

These human in vivo data further the evidence from other species and models that essential genes can be good drug targets. Homozygous knockout of *Hmgcr* and *Ptgs2* are lethal in mice^19–21^. Drug targets exhibit higher inter-species conservation than other genes^22^. Targets of negative drugs include 14 genes with lethal heterozygous knockout mouse phenotypes reported^23^ and 6 reported as essential in human cell culture^24^.

## Prospects for finding human ‘knockouts’

**Fig. 2:**
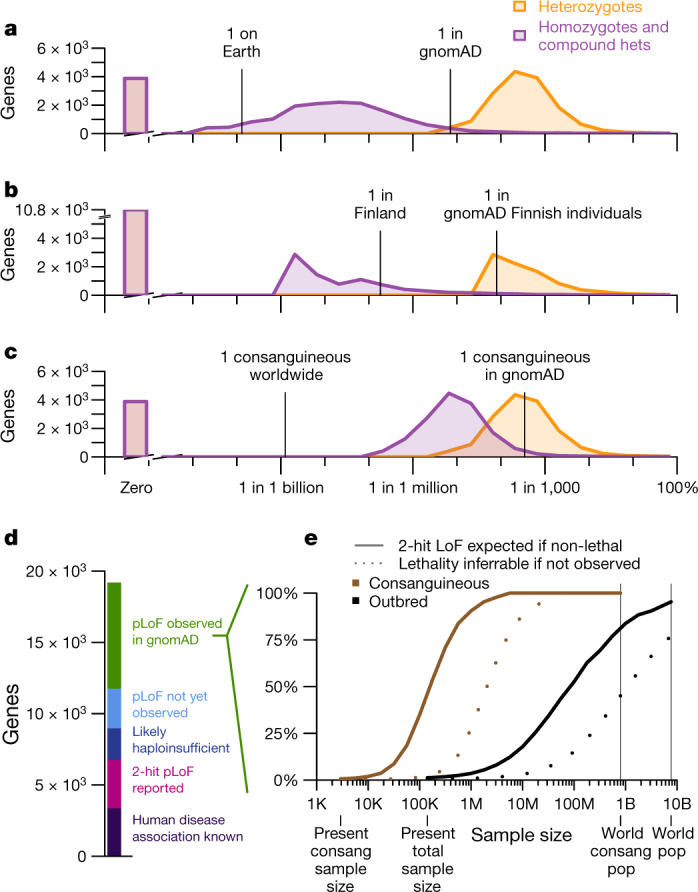
Prospects for discovery of human knockouts. **a**–**c**, Histograms (**a**–**c**): genes by expected heterozygote frequency (orange), and two-hit homozygote and compound heterozygote frequency (purple). **a**, Outbred populations. **b**, Finnish individuals; an example of a bottlenecked population. **c**, Consanguineous individuals. **d**, Current status of pLoF or disease association discovery for all protein-coding genes. **e**, Projected sample sizes required for discovery of two-hit individuals (solid lines) and for statistical inference that a two-hit genotype is lethal if no such individuals are observed (dashed lines), for ‘pLoF observed in gnomAD’ genes (**d**) for consanguineous and outbred individuals.

Athough constraint alone is not adequate to nominate or exclude drug targets, the study of individuals with single hit (heterozygous) or two-hit (‘knockout’) LoF genotypes in a gene of interest can be highly informative about the biological effect of engaging that target^5^. To assess prospects for ascertaining knockout individuals, we computed the cumulative allele frequency (CAF) of pLoF variants in each gene (Methods), and then used this to estimate the expected frequency of two-hit individuals under different population structures (Fig. 2) in the absence of natural selection.

Whereas gnomAD is now large enough to include at least one pLoF heterozygote for most (15,317 out of 19,194; 79.8%) genes, ascertainment of total knockout individuals in outbred populations will require 1,000-fold larger sample sizes for most genes: the median expected two-hit frequency of a gene is just six per billion (Fig. 2a). Even if every human on Earth were sequenced, there are 4,728 genes (24.6%) for which identification of even one two-hit individual would not be expected in outbred populations. Intuitively, because the sample size of gnomAD today is larger than the square root of the world population, variants so far seen in zero or only a few heterozygous individuals are not likely to ever be seen in a homozygous state in outbred populations, except where variants prove common in populations not yet well-sampled by gnomAD.

**Extended Data Fig. 3:**
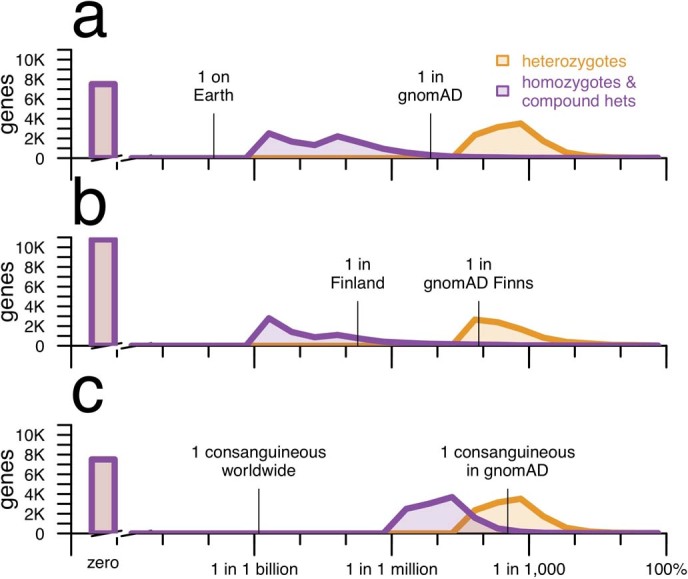
Expected frequency of individuals with one or two null alleles for every protein-coding gene across different population models, with sample size held constant. This is identical to Fig. 2 except as follows. As noted in the Methods, one caveat about Fig. 2 is that the sample size is larger for the plots using all gnomAD exomes (Fig. 2a, c) than for Finnish exomes (Fig. 2b). This figure shows the same analysis, but with the global gnomAD population downsampled to 10,824 randomly chosen exomes so that the same size is identical to that of Finnish exomes. Computation of *P* = 1 − sqrt(*q*) as described in the Methods is computationally expensive for downsampled datasets because it requires individual-level genotypes. Instead, this analysis uses ‘classic’ CAF, which is simply the sum of allele frequencies of all high-confidence pLoF variants each at allele frequency <5%, capped at a total of 100%, for both global and Finnish exomes. The results show that even when the sample size is held constant, the number of genes with zero pLoF variants observed is higher in a bottlenecked population than in a mostly outbred population. A constant *y* axis with no axis breaks is used in this figure to make this difference more clearly visible.

Because population bottlenecks can result in very rare variants present in a founder rising to an unusually high frequency, we also considered knockout discovery in bottlenecked populations, using Finnish individuals in gnomAD as an example^8^. Although this population structure can enable well-powered association studies for the small fraction of genes in which pLoF variants drifted to high frequency due to the bottleneck, overall, identification of two-hit pLoF individuals for a pre-specified gene of interest appears equally or more difficult in Finnish individuals than in outbred populations (Fig. 2b, Extended Data Fig. 3), because rare variants not present in a founder have been effectively removed from the population.

In consanguineous individuals, parental relatedness greatly increases the frequency of homozygous pLoF genotypes. The *n* = 2,912 individuals in the East London Genes & Health (ELGH) cohort^25^ who report having parents who are second cousins or closer have on average 5.8% of their genomes autozygous. Here, the expected frequency of two-hit individuals is many times higher than in outbred populations, at five per million for the median gene (Fig. 2c).

These projections allow us to draft a roadmap for discovery of human knockouts across 19,194 genes (Fig. 2d, e). Online Mendelian Inheritance in Man (OMIM) already describes human disease association for 3,367 genes (18%), although the discovery of LoF individuals in population databases will still be valuable for assessing penetrance and identifying LoF syndromes of known gain-of-function genes. Another 3,421 genes (18%) without known human disease association have two-hit pLoF genotypes reported in gnomAD^7^, ELGH^26^, PROMIS^27^, deCODE^28^ or UK Biobank^29^, which suggests that this genotype may be tolerated. An additional 2,190 genes (11%) appear intolerant of heterozygous inactivation (pLI score > 0.9) in gnomAD—a set expected to be enriched for genes with severe heterozygous and lethal homozygous LoF phenotypes. Another 2,781 genes (14%) have no pLoF variants observed in gnomAD, but our sample size is not yet large enough to robustly infer LoF intolerance. For these genes, observation of outbred two-hit individuals is not expected, and we cannot yet assess the feasibility of identifying consanguineous two-hit individuals because we lack an estimate of pLoF allele frequency.

This leaves 7,435 genes (39%) for which one or more pLoFs are observed in gnomAD, but strong LoF intolerance cannot be determined, two-hit genotypes have not been observed, and a human disease phenotype is not known. We projected the sample sizes required to identify knockout individuals for these genes (Fig. 2e). In outbred populations, current sample sizes would need to increase by approximately 1,000-fold before ascertainment of a single two-hit LoF individual would be expected for the typical gene. By contrast, around a 10- to 100-fold increase from current consanguineous sample size, meaning hundreds of thousands of individuals in absolute terms, would identify at least one two-hit LoF individual for the typical gene. Among other simplifying assumptions (Methods), these projections presume that complete knockout is tolerated. When only one or a few two-hit individuals are expected in a dataset, the absence of any such individuals can be due to either early lethality, a severe clinical phenotype incompatible with inclusion in gnomAD, or simply chance. Thus, the ability to infer lethality of the two-hit genotype based on statistical evidence will lag behind the identification of two-hit individuals where they do exist (Fig. 2e). For some genes, inference of lethality will always remain impossible in outbred populations, though it may be feasible in consanguineous individuals.

## Curation of pLoF variants

**Fig. 3:**
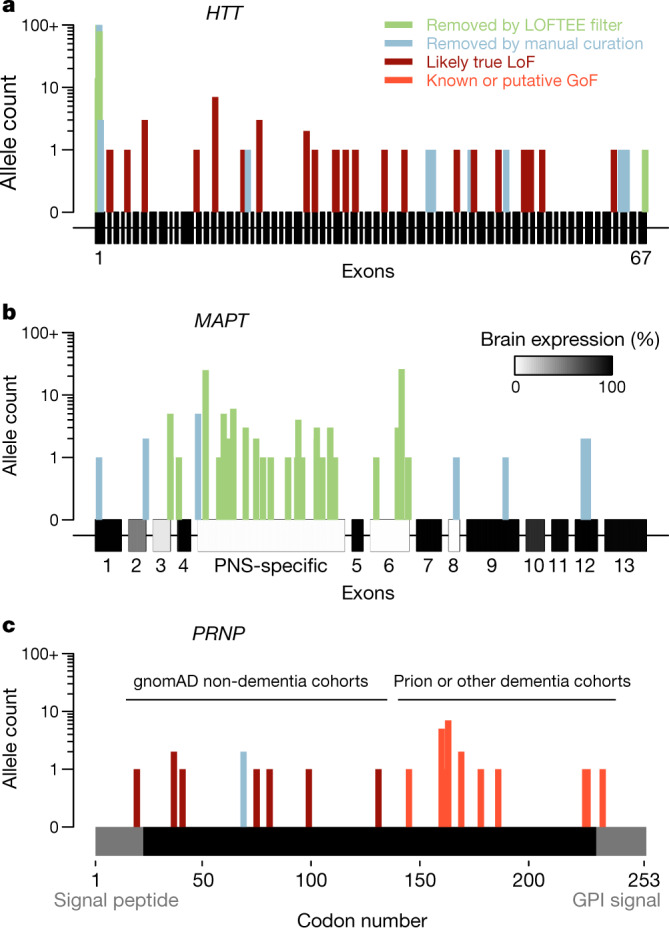
Insights from non-random positional distributions of pLoF variants. **a**–**c**, *HTT* (**a**), *MAPT*, with brain expression data from GTEx^40^ (**b**) and *PRNP*, a single protein-coding exon with domains removed by post-translational modification in grey (**c**), showing previously reported variants^41^ and those newly identified in gnomAD and in the literature (Extended Data Table 5). GPI, glycosylphosphatidylinositol. Detailed variant curation results are provided in Supplementary Table 1.

Where pLoF variants can be identified, they are a valuable resource for assessing the effect of lifelong reduction in gene dosage. To highlight the challenges and opportunities of identifying such variants, we manually curated gnomAD data and the scientific literature for six genes associated with gain-of-function (GoF) neurodegenerative diseases, for which inhibitors or suppressors are under development^30–35^: *HTT* (Huntington's disease), *MAPT* (tauopathies), *PRNP* (prion disease), *SOD1* (amyotrophic lateral sclerosis), and *LRRK2* and *SNCA* (Parkinson's disease). The results (Fig. 3, Extended Data Table 3) illustrate four points about pLoF variant curation.

First, other things being equal, genes with longer coding sequences offer more opportunities for LoF variants to arise, and so tend to have a higher cumulative frequencies of LoF variants, unless they are heavily constrained. Ascertainment of LoF individuals is thus harder for shorter and/or more constrained genes, even though these may be good targets.

Second, many variants annotated as pLoF are false positives^6^, and these are enriched for higher allele frequencies, so that both filtering and curation have an outsized effect on the cumulative allele frequency of LoF. Studies of human pLoF variants lacking stringent curation can therefore easily dilute results with false pLoF carriers.

Third, after careful curation, cumulative LoF allele frequency is sometimes sufficiently high to place certain bounds on what heterozygote phenotype might exist. For example, GoF mutations causing genetic prion disease have a genetic prevalence of approximately 1 in 50,000^36^ and have been known for three decades, with thousands of cases identified, making it unlikely that a comparably severe and penetrant haploinsufficiency syndrome associated with *PRNP* would have gone unnoticed to the present day despite being more than twice as common (roughly 1 in 18,000). Similar arguments can be made for *HTT*, *LRRK2* and *SOD1* genes (Extended Data Tables 3, 4). Of course, this does not rule out a less severe or less penetrant heterozygous LoF phenotype.

Finally, careful inspection of the distributions of pLoF variants can reveal important error modes or disease biology. *HTT*, *MAPT* and *PRNP* genes each have different non-random positional distributions of pLoF variants (Fig. 3). High-frequency *HTT* pLoF variants cluster in the polyglutamine/polyproline repeat region of exon 1 and appear to be alignment artefacts (Fig. 3a). True *HTT* LoF variants are rare and the gene is highly constrained, which might suggest some fitness effect in a heterozygous state in addition to the known severe homozygous phenotype^37,38^, although the frequency of LoF carriers still argues against a penetrant syndromic illness, consistent with the lack of phenotype reported in heterozygotes identified so far^38,39^. High-frequency *MAPT* pLoF variants cluster in exons not expressed in the brain in GTEx data^14,40^, and all remaining pLoFs appear to be alignment or annotation errors (Fig. 3b). No true LoFs are observed in *MAPT*, although our sample size is insufficient to prove that *MAPT* LoF is not tolerated—among constitutive brain-expressed exons, we expect 12.6 LoFs and observe 0, giving a 95% confidence interval upper bound of 23.7% for obs/exp values. *PRNP*-truncating variants in gnomAD cluster in the N terminus; the sole C-terminal truncating variant in gnomAD is a dementia case (Extended Data Table 5), consistent with variants at codon ≥145 causing a pathogenic gain-of-function through change in localization (Fig. 3c). Within codons 1–144, *PRNP* is unconstrained (Extended Data Table 3), and no neurological phenotype has been identified in individuals with truncating variants so far, consistent with the hypothesis that N-terminal truncating variants are true LoF and are tolerated in a heterozygous state^41^.

## Discussion

Studying human gene inactivation can illuminate human biology and guide the selection of drug targets, complementing mouse knockout studies^42^, but analysis of any one gene requires genome-wide context to set expectations and guide inferences. Here we have used gnomAD data to provide context to aid in the interpretation of human LoF variants.

Targets of approved drugs range from highly constrained to completely unconstrained. There may be several reasons why some genes apparently tolerate pharmacological inhibition but not genetic inactivation. LoF variants in constitutive exons should affect all tissues for life, whereas drugs differ in tissue distribution and timing and duration of use. Many drugs known or suspected to cause fetal harm are tolerated in adults^43^, and might target developmentally important genes. Constraint is thought to primarily reflect selection against heterozygotes^13^, the effective gene dosage of which may differ from that achieved by a drug. Constraint measures natural selection over centuries or millennia; the environment of our ancestors presented different selective pressures from what we face today. Actions of small-molecule drugs may not map one-to-one onto genes^44–47^. Regardless, these human in vivo data show that even a highly deleterious knockout phenotype is compatible with a gene being a viable drug target.

For most genes, the lack of total knockout individuals identified so far does not yet provide statistical evidence that this genotype is not tolerated. Indeed, for many genes, such evidence may never be attainable in outbred populations. Bottlenecked populations, individually, are unlikely to yield two-hit individuals for a pre-specified gene of interest, although the sequencing of many different, diverse bottlenecked populations will certainly expand the set of genes accessible by this approach. Identification of two-hit individuals will be most greatly aided by increased investment in consanguineous cohorts, in which the sample size required for any given gene is often orders of magnitude lower than in outbred populations. Our analysis is limited by sample size, insufficient diversity of sampled populations, and simplifying assumptions about population structure and distribution of LoF variants, so our calculations should be taken as rough, order-of-magnitude estimates. Nonetheless, this strategic roadmap for the identification of human knockouts should inform future research investments and rationalize the interpretation of existing data.

Recall-by-genotype efforts are only valuable if the variants in question are correctly annotated. Automated filtering^7^ and transcript expression-aware annotation^14^ are powerful tools, but we demonstrate the continued value of manual curation for excluding further false positives, assessing and interpreting the cumulative allele frequency of true LoF variants, and identifying error modes or biological phenomena that give rise to non-random distributions of pLoF variants across a gene. Such curation is essential before any recontact efforts, and establishing methods for high-throughput functional validation^48^ of LoF variants is a priority. Our curation of pLoF variants in neurodegenerative disease genes is limited by a lack of functional validation and detailed phenotyping; a companion paper demonstrates a deeper investigation of the effects of LoF variants in the *LRRK2* gene^49^.

Drug development projects may increasingly be accompanied by efforts to phenotype human carriers of LoF variants. With the cost of drug discovery driven overwhelmingly by failure^50^, successful interpretation of LoF data to select the right targets and right clinical pathways will yield outsize benefits for research productivity and, ultimately, human health.

## Methods

No statistical methods were used to predetermine sample size. The experiments were not randomized, and investigators were not blinded to allocation during experiments and outcome assessment.

### Data sources

pLoF analyses used the gnomAD dataset of 141,456 individuals^7^. For data consistency, all genome-wide constraint and CAF analyses used only the 125,748 gnomAD exomes. Curated analyses of individual genes used all 141,456 individuals including 15,708 whole genomes. Gene lists used in this study were extracted from public data sources between September 2018 and June 2019. Data sources and criteria for gene list extraction are shown in Extended Data Table 1. This study was performed under ethical approval from the Partners Healthcare Institutional Research Board (2013P001339/MGH) and the Broad Institute Office of Research Subjects Protection (ORSP-3862). All research participants provided informed consent.

### Calculation of pLoF constraint

The calculation of constraint values for genes has been described in general elsewhere^10,12^ and for this dataset specifically by Karczewski et al.^7^. Constraint calculations used LOFTEE-filtered (‘high confidence’) single-nucleotide variants (which for pLoF means nonsense and essential splice site mutations) found in gnomAD exomes with minor allele frequency <0.1%. Only unique canonical transcripts for protein-coding genes were considered, yielding 17,604 genes with available constraint values. For curated genes (Extended Data Table 2), the number of observed variants passing curation was divided by the expected number of variants to yield a curated constraint value. For *PRNP*, the expected number of variants was adjusted by multiplying by the ratio of the sum of mutation frequencies for all possible pLoF variants in codons 1–144 to the sum of mutation frequencies for all possible pLoF variants in the entire transcript, yielding 6 observed out of 6.06 expected. For *MAPT*, the expected number of variants was taken from Ensembl transcript ENST00000334239, which includes only the exons identified as constitutively brain-expressed in Fig. 3b (exon numbering previously described^51^).

### Calculation of pLoF heterozygote and homozygote/compound heterozygote frequencies

LOFTEE-filtered high-confidence pLoF variants with minor allele frequency <5% in 125,748 gnomAD exomes were used to compute the proportion of individuals without a loss-of-function variant (*q*); the CAF was computed as *p* = 1 − sqrt(*q*). This approach conservatively assumes that, if an individual has two different pLoF variants, they are in *cis* to each other and count as only one pLoF allele.

For outbred populations (Fig. 2a), we used the value of *p* from all 125,748 gnomAD exomes, as this allows the largest possible sample size. This includes some individuals from bottlenecked populations, for which the distribution of *p* does differ from outbred populations, but these individuals are a small proportion of gnomAD exomes (12.6%). This also includes some consanguineous individuals, but these are an even smaller proportion of gnomAD exomes (2.3%), and any difference in the value of *p* between consanguineous and outbred populations is expected to be very small. Heterozygote frequency was calculated as 2*p*(1 −*p*) and homozygote and compound heterozygote frequency was calculated as *p*^2^. Lines indicate the size of gnomAD (141,456 individuals) and the world population (6.69 billion).

For bottlenecked populations (Fig. 2b), we used the value of *p* from the 10,824 Finnish exomes only. Lines indicate the number of Finnish individuals in gnomAD (12,526) and the population of Finland (5.5 million).

For consanguineous individuals (Fig. 2c), we again used the value of *p* from all gnomAD exomes, because *p* is not expected to differ greatly in consanguineous versus outbred populations. We used the mean proportion of the genome in runs of autozygosity (*a*) from individuals self-reporting second cousin or closer parents in East London Genes & Health, *a* = 0.05766 (rounded to 5.8%). Heterozygote frequency was calculated as 2*p*(1 − *p*) and homozygote and compound heterozygote frequency was calculated as (1 − a)*p*^2^ + *ap*. Lines indicate the number of consanguineous South Asian individuals in gnomAD (*n* = 2,912, by coincidence the same number as report second cousin or closer parents in ELGH) based on *F* > 0.05 (a conservative estimate, because second cousin parents are expected to yield *F* = 0.015625), and the estimated number of individuals in the world with second cousin or closer parents (10.4% of the world population)^9^.

Several caveats apply to our CAF analysis. First, our approach naively treats genes with no pLoFs observed as having *P* = 0, even though pLoFs might be discovered at a larger sample size. Second, we naively group all populations together, even though the distribution of populations sampled in gnomAD does not reflect the world population^7^; we believe that this is reasonable because CAF for many genes is driven by singletons and other ultra-rare variants for which frequency is not expected to differ appreciably by continental population^10^. (It is important to note that the histograms shown in Fig. 2 reflect the expected frequency of heterozygotes and homozygotes/compound heterozygotes, based on gnomAD allele frequency, rather than the actual observed frequency of individuals with these genotypes in gnomAD.) Third, we use only protein-truncating variants annotated as pLoF in gnomAD. Structural and non-coding variation resulting in a loss of function may be missed in exomes, and missense variants resulting in a loss of function cannot be rigorously annotated. Fourth, we naively treat genes with one pLoF allele observed as having *P* = 1/(2 × 125,748), even though on average singleton variants have a true allele frequency lower than their nominal allele frequency^10^. Fifth, the variants included in this analysis are filtered but have not been manually curated or functionally validated, so some will ultimately prove not to be true LoF. These false positives tend to be more common and will have disproportionately contributed to the cumulative LoF allele frequency. Sixth, as described in the main text, our calculations assume that complete knockout is tolerated, which will not be true for some genes. We therefore also include a projection of the sample size needed to infer lethality from the absence of two-hit knockout individuals (Fig. 2e). Points one to three will tend to lead to underestimation of the true complete knockout frequency, whereas points four to six will tend to lead to overestimation. On balance, our calculations may reflect an upper bound of complete knockout frequency for most genes owing to the strong influence of factors five and six. Finally, as a matter of comparison between population structures, the sample size for all gnomAD exomes (Fig. 2a, c) is larger than for only Finnish exomes (Fig. 2b). For a version of Fig. 2 with the global gnomAD population downsampled to the same sample size as the gnomAD Finnish population, see Extended Data Fig. 2.

### Knockout roadmap

For the knockout ‘roadmap’ (Fig. 2d, e), we classified genes according to the current status of human disease association and LoF ascertainment. Genes were classified as having a Mendelian disease association if they were present in OMIM with the filters described in Extended Data Table 1.

Remaining genes were classified as ‘2-hit LoF reported’ based on presence in one or more of the following gene lists: homozygous LoF genotypes in gnomAD curated as previously described^7^; filtered homozygous LoF genotypes in runs of autozygosity with minor allele frequency <1% in canonical transcripts in the Bradford, Birmingham and ELGH^25^ cohorts (total *n* = 8,925); observed number of imputed homozygotes >1 or number of compound heterozygous carriers where minor allele frequency <2% (for both variants) in deCODE^28^; homozygous LoF reported in PROMIS^27^; homozygous LoF with minor allele frequency <1% in UK Biobank^29^.

The remainder of genes were sequentially classified as ‘likely haploinsufficient’ if pLI >0.9 in gnomAD, ‘pLoF not yet observed’ if CAF = 0 in gnomAD, and, finally, ‘pLoF observed in gnomAD’ if CAF >0 in gnomAD.

### Genetic prevalence estimation

Here, we define ‘genetic prevalence’ for a given gene as the proportion of individuals in the general population at birth who have a pathogenic variant in that gene that will cause them to later develop disease. Genetic prevalence has not been well-studied or estimated for most disease genes.

In principle, it should be possible to estimate genetic prevalence simply by examining the allele frequency of reported pathogenic variants in gnomAD. In practice, three considerations usually preclude this approach. First, the present gnomAD sample size of 141,456 exomes and genomes is still too small to permit accurate estimates for very rare diseases. Second, the mean age of gnomAD individuals is approximately 55, which is above the age of onset for many rare genetic diseases, and individuals with known Mendelian disease are deliberately excluded, so pathogenic variants will be depleted in this sample relative to the whole birth population. Third and most importantly, a large fraction of reported pathogenic variants lack strong evidence for pathogenicity and are either benign or low penetrance^10,41^, so without careful curation of pathogenicity assertions, summing the frequency of reported pathogenic variants in gnomAD will in most cases vastly overestimate the true genetic prevalence of a disease.

Instead, we searched the literature and very roughly estimated genetic prevalence based on available data. In most cases, we took disease incidence (new cases per year per population), multiplied by proportion of cases due to variants in a gene of interest, and multiplied by average age at death in cases. In some cases, estimates of at-risk population or direct measures of genetic prevalence were available. Details of the calculations undertaken for each gene are provided in Extended Data Table 4.

### Reporting summary

Further information on research design is available in the Nature Research Reporting Summary linked to this paper.

## Online content

Any methods, additional references, Nature Research reporting summaries, source data, extended data, supplementary information, acknowledgements, peer review information; details of author contributions and competing interests; and statements of data and code availability are available at 10.1038/s41586-020-2267-z.

## Supplementary information

Reporting Summary

Supplementary Table 1 This supplementary table contains details of curated variants in neurodegenerative disease genes.

Peer Review File Reviewer reports and authors’ response from the peer review of this Article at Nature.

## Figures and Tables

**Extended Data Table 1:**
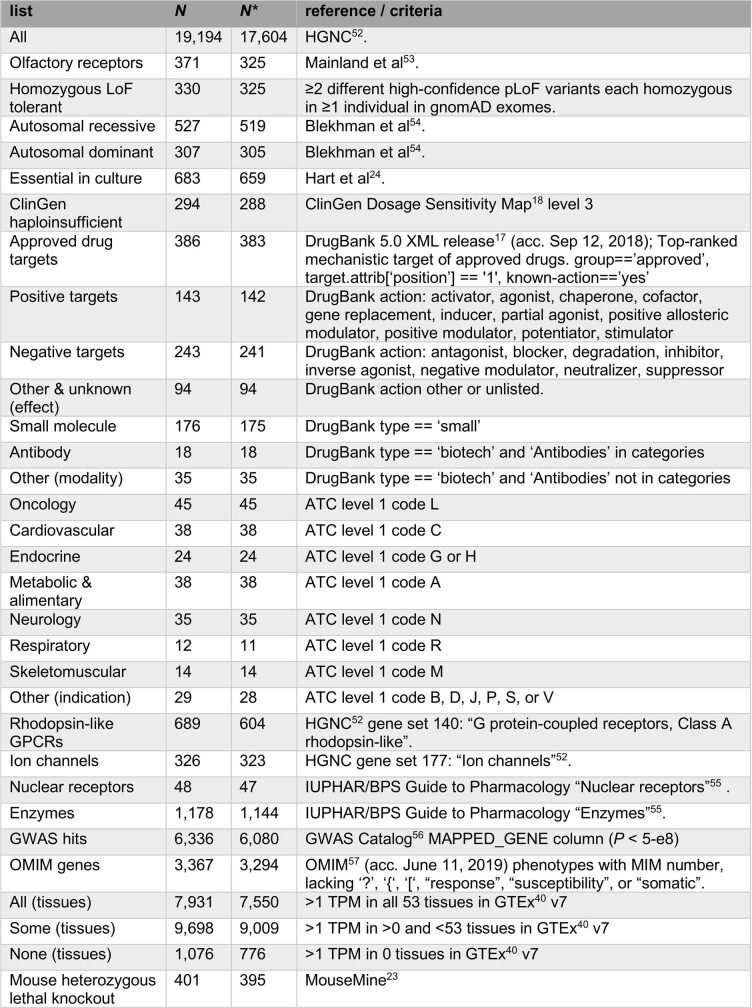
Data sources for gene lists used in this study

For analysis, only protein-coding genes with unambiguous mapping to current approved gene symbols were used; numbers in the table reflect this. Values in the *N* column indicate totals from the full universe of 19,194 genes; values in the N* column indicate the subset of genes with non-missing constraint values, used for Fig. 1 and Extended Data Figs. 1, 2. The following references are cited in the table: refs. ^17,18,23,24,40,52–57^.

**Extended Data Table 2:**
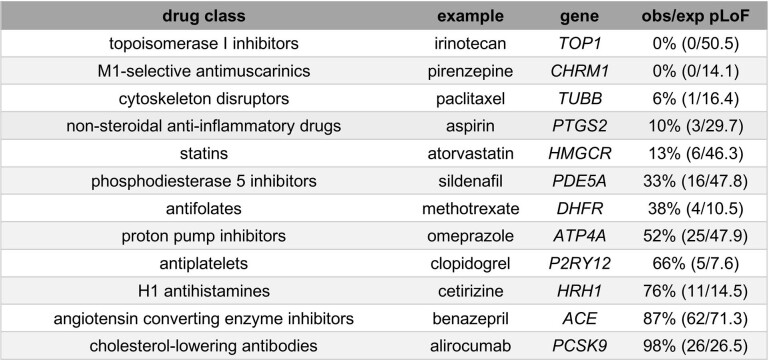
Spectrum of tolerance to genetic inactivation among human drug targets

Example targets are arranged from the most intolerant (top) to the most tolerant (bottom) of inactivation.

**Extended Data Table 3:**
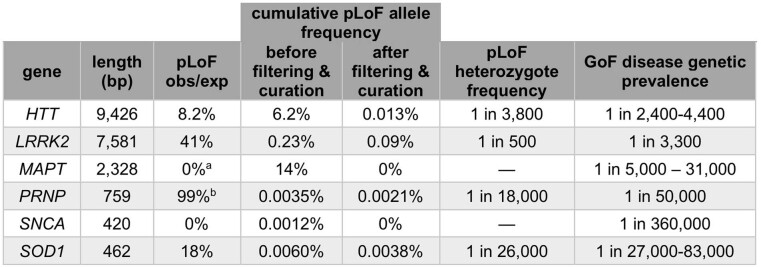
Curation of pLoF variation in six neurodegenerative disease genes

Shown are the coding sequence length (base pairs, bp), constraint value (pLoF obs/exp) after filtering and curation, cumulative allele frequency before and after filtering and manual curation, estimated frequency of true pLoF heterozygotes in the population, and genetic prevalence (population frequency including pre-symptomatic individuals) of the GoF disease associated with the gene. Genetic prevalence calculations are described in Extended Data Table 4, and variant curation details are provided in Supplementary Table 1, except for *LRRK2*, which is described in detail elsewhere^49^.

^a^Constitutive brain-expressed exons only.

^b^*PRNP* codons 1–144; see Fig. 3c for rationale.

**Extended Data Table 4:**
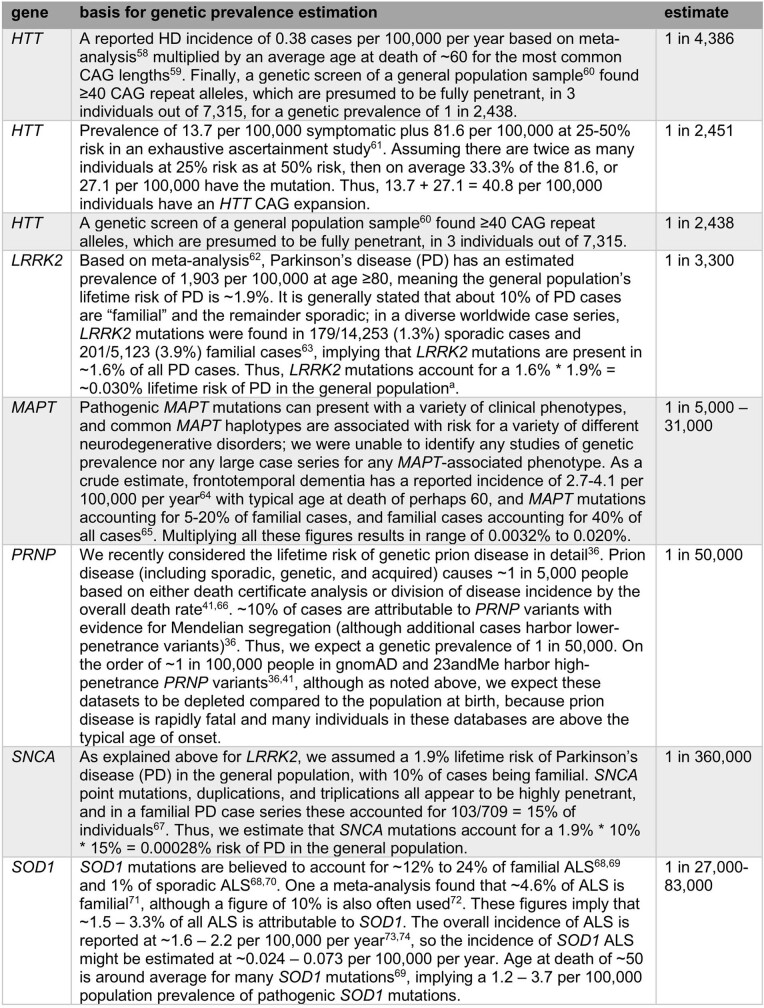
Estimation of genetic prevalence for GoF genetic neurodegenerative diseases

Data sources were identified by PubMed and Google Scholar searches. Genetic prevalence was defined as the proportion of the population at birth carrying a mutation and destined to later develop disease, and estimated as described for each gene. The following references are cited in the table: refs. ^36,41,58–74^.

^a^It is important to consider how this figure relates to the penetrance of *LRRK2* mutations, as *LRRK2* variants appear to occupy a spectrum of penetrance^75^. Some variants exhibit Mendelian segregation with disease^76,77^, implying high risk; the G2019S variant is estimated to have approximately 32% penetrance^78^; and other common variants are risk factors with odds ratios of only around 1.2 estimated through genome-wide association studies (GWAS)^79^. The GWAS-implicated common variants were not included in the case series on which our estimate is based^63^, but G2019S does account for most cases in that series. Because the 0.03% estimate here is based on counting symptomatic cases rather than asymptomatic individuals, it will appropriately underestimate the number of G2019S carriers. In essence, in this calculation each G2019S carrier in the population only counts as 1/3 of a person, because they have only a 1/3 probability of developing a disease. It is therefore appropriate that our estimate of genetic prevalence (0.03%) is actually lower than double the allele frequency of G2019S in gnomAD (0.1%).

**Extended Data Table 5:**
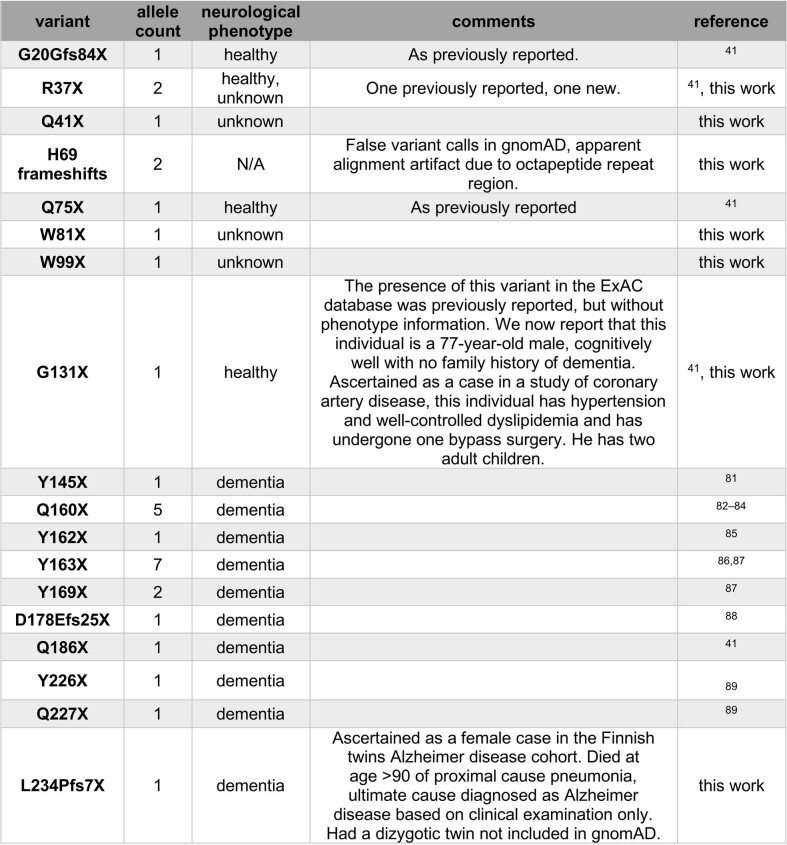
Details of *PRNP*-truncating variants

Allele count for variants from the literature in Fig. 3c is the total number of definite or probable cases with sequencing performed in the studies cited in this table. The L234Pfs7X variant changes the C-terminal GPI signal of prion protein from SMVLFSSPPVILLISFLIFLIVGX to SMVPSPLHLX. This new sequence does not adhere to the known rules of GPI anchor attachment^80^: GPI signals must contain a 5–10-polar-residue spacer followed by 15–20 hydrophobic residues. Thus, this frameshifted prion protein would be predicted to be secreted and thus may be pathogenic, explaining the Alzheimer’s disease diagnosis in this individual. However, it is also possible that the new C-terminal sequence found here interferes with prion formation, and/or that this variant is incompletely penetrant, and that the diagnosis of Alzheimer’s disease in this individual is merely a coincidence. The following references are cited in the table: refs. ^41,81–89^.

## Data Availability

The gnomAD v2 data are available via the gnomAD browser (https://gnomad.broadinstitute.org).

## Code availability

Additional data and the R 3.5.1 and Python 2.7.10 source code for this study are available via GitHub (https://github.com/ericminikel/drug_target_lof).
