# Supplementary material for: Evaluating drug targets through human loss-of-function genetic variation
Source: Nature. 2020 May 27;581(7809):459–64. doi: 10.1038/s41586-020-2267-z (PMC7272226; doi:10.1038/s41586-020-2267-z)
Supplement: Supplementary file 1 — Reporting Summary [file 41586_2020_2267_MOESM1_ESM.pdf]

nature research

Corresponding author(s): Eric Vallabh Minikel and Daniel G. MacArthur

Last updated by author(s): 11/13/2019

Reporting Summary

Nature Research wishes to improve the reproducibility of the work that we publish. This form provides structure for consistency and transparency in reporting. For further information on Nature Research policies, see Authors & Referees and the Editorial Policy Checklist.

Statistics

For all statistical analyses, confirm that the following items are present in the figure legend, table legend, main text, or Methods section.

n/a	Confirmed	
☐	☒	The exact sample size (n) for each experimental group/condition, given as a discrete number and unit of measurement
☒	☐	A statement on whether measurements were taken from distinct samples or whether the same sample was measured repeatedly
☐	☒	The statistical test(s) used AND whether they are one- or two-sided<br>*Only common tests should be described solely by name; describe more complex techniques in the Methods section.*
☐	☒	A description of all covariates tested
☐	☒	A description of any assumptions or corrections, such as tests of normality and adjustment for multiple comparisons
☐	☒	A full description of the statistical parameters including central tendency (e.g. means) or other basic estimates (e.g. regression coefficient) AND variation (e.g. standard deviation) or associated estimates of uncertainty (e.g. confidence intervals)
☐	☒	For null hypothesis testing, the test statistic (e.g. F, t, r) with confidence intervals, effect sizes, degrees of freedom and P value noted<br>*Give P values as exact values whenever suitable.*
☒	☐	For Bayesian analysis, information on the choice of priors and Markov chain Monte Carlo settings
☒	☐	For hierarchical and complex designs, identification of the appropriate level for tests and full reporting of outcomes
☒	☐	Estimates of effect sizes (e.g. Cohen's d, Pearson's r), indicating how they were calculated

Our web collection on statistics for biologists contains articles on many of the points above.

Software and code

Policy information about availability of computer code

Data collection	Analyses utilized Python 2.7.10 and R 3.5.1. Data and code sufficient to produce the plots and analyses in this paper are available at https://github.com/ericminikel/drug_target_lof
Data analysis	Analyses utilized Python 2.7.10 and R 3.5.1. Data and code sufficient to produce the plots and analyses in this paper are available at https://github.com/ericminikel/drug_target_lof

For manuscripts utilizing custom algorithms or software that are central to the research but not yet described in published literature, software must be made available to editors/reviewers. We strongly encourage code deposition in a community repository (e.g. GitHub). See the Nature Research guidelines for submitting code & software for further information.

Data

Policy information about availability of data

All manuscripts must include a data availability statement. This statement should provide the following information, where applicable:

- Accession codes, unique identifiers, or web links for publicly available datasets
- A list of figures that have associated raw data
- A description of any restrictions on data availability

Analyses utilized Python 2.7.10 and R 3.5.1. Data and code sufficient to produce the plots and analyses in this paper are available at https://github.com/ericminikel/drug_target_lof

Field-specific reporting

Please select the one below that is the best fit for your research. If you are not sure, read the appropriate sections before making your selection.

☒ Life sciences ☐ Behavioural & social sciences ☐ Ecological, evolutionary & environmental sciences

For a reference copy of the document with all sections, see nature.com/documents/nr-reporting-summary-flat.pdf

Life sciences study design

All studies must disclose on these points even when the disclosure is negative.

Sample size	This study was opportunistic, and involved secondary use of all available genome and exome data. No sample size was predetermined. Our flagship analysis of gnomAD loss-of-function variants (Karczewski et al, https://doi.org/10.1101/531210) indicates that the dataset is well-powered to examine constraint against such variants — for instance, 72% of genes have at least 10 pLoF variants expected in this sample size based on mutation rates.
Data exclusions	Sample QC and variant QC for the gnomAD database are described extensively by Karczewski et al, https://doi.org/10.1101/531210. Notably, individuals with severe pediatric disease, and known first disease relatives of those with severe pediatric disease were excluded.
Replication	We did not attempt to reproduce any findings in a separate dataset, as no other exome or genome sequencing dataset of comparable size exists.
Randomization	As this was a population-based study, and not a case-control study, no randomization was performed.
Blinding	As this was a population-based study, and not a case-control study, blinding was not relevant.

Reporting for specific materials, systems and methods

We require information from authors about some types of materials, experimental systems and methods used in many studies. Here, indicate whether each material, system or method listed is relevant to your study. If you are not sure if a list item applies to your research, read the appropriate section before selecting a response.

Materials & experimental systems

n/a	Involved in the study	
☒	☐	Antibodies
☒	☐	Eukaryotic cell lines
☒	☐	Palaeontology
☒	☐	Animals and other organisms
☐	☒	Human research participants
☒	☐	Clinical data

Methods

n/a	Involved in the study	
☒	☐	ChIP-seq
☒	☐	Flow cytometry
☒	☐	MRI-based neuroimaging

Human research participants

Policy information about studies involving human research participants

Population characteristics	As an opportunistic collection of data, the participants in gnomAD were not selected based on age, gender, or genotypic information. As described above, individuals with severe pediatric disease, and known first disease relatives of those with severe pediatric disease were excluded. The population and dataset inclusion criteria are described in more detail by Karczewski et al, https://doi.org/10.1101/531210
Recruitment	The generation of the gnomAD database was an opportunistic secondary use study, we did not recruit any participants. The study is described in more detail by Karczewski et al, https://doi.org/10.1101/531210
Ethics oversight	This study was performed under ethical approval from the Partners Healthcare Institutional Research Board (2013P001339/MGH) and the Broad Institute Office of Research Subjects Protection (ORSP-3862) in compliance with all relevant ethical regulations; informed consent was obtained from all research participants.

Note that full information on the approval of the study protocol must also be provided in the manuscript.
