# Supplementary material for: Evaluating drug targets through human loss-of-function genetic variation
Source: Nature. 2020 May 27;581(7809):459–64. doi: 10.1038/s41586-020-2267-z (PMC7272226; doi:10.1038/s41586-020-2267-z)
Supplement: Supplementary file 3 — Reviewer reports and authors’ response from the peer review of this Article at Nature. [file 41586_2020_2267_MOESM3_ESM.pdf]

natureresearch

Peer Review File

Manuscript Title: Analyses: Evaluating drug targets through human loss-of-function genetic variation

Reviewer Comments & Author Rebuttals

Reviewer Reports on the Initial Version:

Referee #1 (Remarks to the Author):

Summary

The current study examines the utility of predicted loss-of-function (pLoF) variation for the evaluation of genes as potential drug targets. Using gnomAD database of 141,456 human genomes and exomes, they categorize variants as pLoF if such variants were annotated as nonsense, frameshift, or essential splice site-disrupting (aka, protein-truncating variants). They then use the pLoF metric in three different sets of analyses to inform on drug target success.

First (constraint as predictor of drug target success), they use the obs/exp constraint metric for pLoF variants (see Figure 1) to assess the degree of natural selection against loss-of-function variants in the targets of approved drugs (n=383). They found that drug targets are on average more depleted for pLoF variation than other genes (Fig. 2a), even when stratified by the drug's effect on its target (Fig. 2b). Next, the study sought to identify potential confounding variables (e.g., “druggable” targets, human genetic validation, tissue expression) that could explain the finding that drug targets are more depleted for pLoF variation than other genes. They found that canonically "druggable" targets have LoF obs/exp ratios significantly different from the set of all genes (Figure 3). They found that neither genetic disease association nor tissue expression could explain the skewed distribution. The authors conclude: “Thus, although drug targets are more constrained than the average gene even after controlling for the variables considered here, it would not necessarily be appropriate to conclude that stronger pLoF constraint is associated with increased likelihood of drug target success.”

Second (ascertainment of human knockouts), the study examines the prospects for ascertainment of heterozygous or homozygous “knockout” humans for target validation. They computed the cumulative allele frequency of pLoF variants in each gene in gnomAD in order to assess how often heterozygous or homozygous null individuals might be identified across different populations (Fig. 4). They conclude that outbred populations alone (Fig. 4a) will not be sufficient to identify human knockouts, and that special populations (e.g., bottleneck [Fig. 4b], consanguineous [Fig. 4c]) will be required. Of note, bottleneck populations have the unique property that a small number of “jackpot” genes will harbor common pLoF variants, with most other genes harboring few or no rare pLoF variants, as these variants did not pass through the bottleneck.

Third (curation of true LoF variants in six neurodegenerative genes), the study deeply investigates six genes that harbor gain-of-function (GoF) mutations that lead to neurodegenerative diseases in order to curate the world’s data on true LoF variants (predicted, disease-associated, etc.). They find that “many variants annotated as pLoF are in fact false positives, and this is particularly true of pLoF variants with higher allele frequencies,” (Table 2). Going further, they demonstrate that the “positional distribution of pLoF variants often appears non-random”, which they illustrate for 3 genes (Fig. 5). Despite these limitations of pLoF variants, the study suggests, based on cumulative frequency of pLoF variants, that it is possible to rule out that a “comparably severe and penetrant heterozygous loss-of-function syndrome associated with the same gene could have gone unnoticed to the present day”. By inference, they suggest that 50% loss-of-function via pharmacologic manipulation should be tolerated in humans.

Fourth (recommendations), based on the above findings, the study offers recommendations for the use of human knockouts for drug target evaluation (Box 1): filter and curate pLoF variants; estimate whether it is possible to ascertain homozygous null “human knockouts”; validate loss-of-function experimentally; and do not use constraint alone as a predictor of drug target success.

Critique

The major findings are: (1) while drug targets demonstrate more constraint than the average gene in the human genome,

constraint alone is unlikely to be useful in selecting drug targets; (2) the distribution of pLoF variants can be used to predict the number of genes that are likely to harbor homozygous null mutations (i.e., human knockouts) across outbred and special populations; and (3) many predicted LoF variants are likely false positives, and these false positives have a non-random distribution across genes. Based on these findings, the study provides recommendations for the use of human knockouts for drug target evaluation (Box 1).

Overall, I find the topic of great interest, and I believe that the gnomAD dataset provides a very unique resource to answer questions that were previously difficult to address. They test, for the first time, the value of constraint on drug target success. Their approach is scientifically rigorous, but their conclusions are underwhelming: constraint alone is not a strong predictor of drug target success (see Major Comment #1). They also provide, for the first time, a quantitative assessment of the feasibility of identifying homozygous null mutations for every gene in the genome. I find this result potentially very interesting, and I would like to see the authors provide a more quantitative roadmap of a human knockout project, as well as an online resource that allows investigators to explore feasibility for every gene in the genome (Major Comment #2). Finally, the six use cases highlight the importance of curation to minimize false positive findings. This finding raises concerns about the conclusions derived on constraint and ascertainment (Major Comment #3).

Major comments

1. Utility of constraint metric. I am struggling to put these results in context. On one hand, this has been a lingering question in the field which the study addresses in a rigorous manner. On the other hand, the results are less than conclusive – constraint may matter, but it may not. There certainly is value in performing the analysis and reporting the results. But the results are unlikely to be highly impactful for drug discovery.

2. Roadmap for human knockouts. I would like the authors to be more be more quantitative with the number of genes expected to harbor homozygous loss-of-function mutations. There should be a way to integrate data from disease-causing mutations, outbred populations, and special populations (e.g., bottleneck, consanguineous) to arrive at a roadmap for a human knockout project. For example, how many genes have human knockouts that lead to severe disease in humans? For the remainder of genes, how many have been identified in extant human knockout databases (e.g., Iceland, PROMISE, ELGH) as harboring homozygous null mutations? Therefore, how many genes have yet to be identified as having homozygous null mutations? Extrapolating from data shown in Figure 4, how many individuals are required to from outbred and special populations to obtain human knockouts for 25%, 50%, and 75% of genes in the human genome? Going further, it should be possible to create an online portal that would allow investigators to estimate the probability of finding human knockouts for every gene in the genome. This could be an interactive portal that would allow users to input number of genomes sequence across different outbred and special populations.

3. Curation. Their initial conclusions (constrain, ascertainment) are based on unfiltered pLoF variants. However, in the third section of their study on six neurodegenerative disease genes, they conclude that many predicted LoF variants are false positives. How might these false positives impact their conclusions on constraint and human knockout ascertainment?

Minor comments

1. The Abstract provides more background than a summary of the findings in the study, and the Introduction provides a lot of background that is more relevant for a review article. Similarly, the other sections (e.g., constraint, ascertainment, curation) are a bit verbose. Thus, the manuscript could benefit from a more focused writing style throughout.

2. The Introduction claims that a “systematic framework for applying human genetic data to the selection of drug targets and to the prediction of drug safety is lacking” (lines 125-127). I believe this is an exaggerated claim, as several nice reviews have been published on this very topic.

3. Lines 198-199 state that the “overall distribution of pLoF obs/exp values for drug targets was similar to that for all genes”, but line 253 states “drug targets are on average more depleted for pLoF variation than other genes”. These statements appear to contradict each other. Please clarify.

4. Lines 454-456 state “we anticipate that high-throughput direct functional validation of candidate pLoF variants will become the standard for such studies in humans”. It would be useful to provide published references to such methodologies.

Referee #2 (Remarks to the Author):

There's a lot of interest in how human genetics can be exploited to guide drug discovery, in particular for identification and validation of novel targets. The authors have assembled the largest sequencing data set to date to get a systematic and comprehensive view on ultra-rare DNA sequence variation. The specific focus of this paper is on predicted loss-of-function variants that result in human genetic “knockouts” for a gene of interest. The ability to relate such pLoF variants to phenotypes is of great interest: not only for probing the efficacy of gene/protein inhibition with respect to a disease phenotype, but also for assessing potential safety issues for such inhibition. The authors should be congratulated for their intense drive to pull multiple sequencing data sets together in the way that they have, and to create a fantastic open resource that will allow the community to study a number of very fundamental questions in human genetics.

Although the manuscript is a joy to read (as the authors are careful to walk the reader through some of the intuition or logic of what might be expected in light of evolution and selection), the overall message is one where there’s no simple rule to translate the pLoF statistics within a given gene into a meaningful “recommendation” that informs on whether a gene is an efficacious and safe drug target. Despite the fact that there is a statistically significant relative depletion of pLoFs in gene targets of approved drugs (line 212), the overall variation between drug targets (e.g. Table 1) precludes a “simple” rule. In this sense, the intuition shared by the authors (e.g. lines 191-196; lines 224-225; lines 243-249) is not incredibly helpful to rationalize the presented findings.

Also, the idea that bottlenecked and consanguineous populations may boost power for the characterization of pLoF variants is not so novel anymore, nor is the notion that careful QC filtering and curation is absolutely critical to build such databases. I found the example of the six neurodegenerative genes only mildly useful. The idea that different exons may have different biological functions is obvious, and it follows that the distribution of pLoFs and their relative impact will simply mirror that.

Overall, it feels as though the key messages (e.g. Box 1) could have been better presented in a much more condensed format (perhaps as part of the main paper describing gnomAD). As the authors suggest, “the types of analysis described (…) are only a first step” (lines 557-558).

Referee #3 (Remarks to the Author):

In this paper, Minikel et al. analyze the relevance of metrics of gene essentiality to the selection of drug targets for therapeutic development. A first observation is that the distribution of the pLoF metric for drug targets differs from that of all genes, significantly, but not necessarily meaningfully. In other words, gene essentiality is concluded to not be a strong predictor of whether or not the protein product of a given gene will make a good drug target. A second observation is that LoF mutations are infrequent enough that in outbred populations, “human knockouts” will be vanishingly rare to nonexistent, supporting the case for studying such individuals in consanguineous populations. A third observation is that manual curation of pLoF variants in selected genes associated with neurodegenerative disease reveals challenges related to artifacts, positional distribution, etc. that complicate their interpretation. The collective set of observations are used to provide some guidance for the field in assessing the relevance of pLoF mutations to drug target selection.

Overall, this paper is fine in the sense that I don’t think that I take strong issue with anything that is said or the analyses done. However, to be frank I found the degree of novelty to be limited. The authors are appropriately conservative in their conclusions, for example not overly interpreting the rather subtle differences in pLoF distributions for drug targets vs. all genes. At the same time, I struggle to identify what is sufficiently new and compelling here that would warrant publication of this paper in Nature. That PCSK9 LoF mutations are well known to be oddly high in frequency -- it was always a bit of a "winner's curse" outlier -- such that knockouts are discoverable, as is the fact that double knockouts for other genes will be very hard to find in outbred populations, and hard-but-not-impossible to find in consanguineous populations. The distribution of pLoF mutations of drug targets is essentially a negative result – a similar result in some sense to the picture that emerges from simply looking at evolutionary conservation (see the overlapping box plot distributions in Fig 2B of https://www.ncbi.nlm.nih.gov/pmc/articles/PMC4826257/). The authors are appropriately conservative in interpreting the

moderate difference, which is commendable, but the conclusion (which I agree with) is still that the information (pLOF metrics) is of modest to little value, which somewhat undermines the guidance at the end of the paper (i.e. if knowledge of LoF mutations is not that useful for target selection, then why are we worried about drug companies running afoul of artifacts?). On both of the above points (finding double-knockouts to gauge safety and using essentiality to pick targets), the authors may be trying to push back against the field overinterpreting the PCSK9 example, but that's really just the field's own fault for being prone to winner's curse rather than a view that's grounded in a credible set of arguments. The arguments about human knockouts and the fact that they won't be found in outbred populations is correct, but this fact is well predicted by the MAF of LOF mutations and Hardy Weinberg equilibrium. On the last set of observations, regarding challenges in interpreting LoF mutations in databases like ExAC -- again, this is useful, but these kinds of artifacts have been well characterized by the authors and others before, and I'm having trouble finding sufficient novelty here.

Author Rebuttals to Initial Comments:

We thank the Editors and Reviewers for thoughtfully reading and commenting on our manuscript. We have been able to address almost all of the points raised, and are pleased that these have improved the manuscript.

Referee #1 (Remarks to the Author):

Summary

The current study examines the utility of predicted loss-of-function (pLoF) variation for the evaluation of genes as potential drug targets. Using gnomAD database of 141,456 human genomes and exomes, they categorize variants as pLoF if such variants were annotated as nonsense, frameshift, or essential splice site-disrupting (aka, protein- truncating variants). They then use the pLoF metric in three different sets of analyses to inform on drug target success.

First (constraint as predictor of drug target success), they use the obs/exp constraint metric for pLoF variants (see Figure 1) to assess the degree of natural selection against loss-of-function variants in the targets of approved drugs (n=383). They found that drug targets are on average more depleted for pLoF variation than other genes (Fig. 2a), even when stratified by the drug's effect on its target (Fig. 2b). Next, the study sought to identify potential confounding variables (e.g., "druggable" targets, human genetic validation, tissue expression) that could explain the finding that drug targets are more depleted for pLoF variation than other genes. They found that canonically "druggable" targets have LoF obs/exp ratios significantly different from the set of all genes (Figure 3). They found that neither genetic disease association nor tissue expression could explain the skewed distribution. The authors conclude: "Thus, although drug targets are more constrained than the average gene even after controlling for the variables considered here, it would not necessarily be appropriate to conclude that stronger pLoF constraint is associated with increased likelihood of drug target success."

Second (ascertainment of human knockouts), the study examines the prospects for ascertainment of heterozygous or homozygous "knockout" humans for target validation. They computed the cumulative allele frequency of pLoF variants in each gene in gnomAD in order to assess how often heterozygous or homozygous null individuals might be identified across different populations (Fig. 4). They conclude that outbred populations alone (Fig. 4a) will not be sufficient to identify human knockouts, and that special populations (e.g., bottleneck [Fig. 4b], consanguineous [Fig. 4c]) will be

required. Of note, bottleneck populations have the unique property that a small number of"jackpot"

genes will harbor common pLoF variants, with most other genes harboring few or no rare pLoF variants, as these variants did not pass through the bottleneck.

Third (curation of true LoF variants in six neurodegenerative genes), the study deeply investigates six genes that harbor gain-of-function (GoF) mutations that lead to neurodegenerative diseases in order to curate the world's data on true LoF variants (predicted, disease-associated, etc.). They find that "many variants annotated as pLoF are in fact false positives, and this is particularly true of pLoF variants with higher allele frequencies," (Table 2). Going further, they demonstrate that the "positional distribution of pLoF variants often appears non-random", which they illustrate for 3 genes (Fig. 5). Despite these limitations of pLoF variants, the study suggests, based on cumulative frequency of pLoF variants, that it is possible to rule out that a "comparably severe and penetrant heterozygous loss-of-function syndrome associated with the same gene could have gone unnoticed to the present day". By inference, they suggest that 50% loss-of- function via pharmacologic manipulation should be tolerated in humans.

Fourth (recommendations), based on the above findings, the study offers recommendations for the use of human knockouts for drug target evaluation (Box 1): filter and curate pLoF variants; estimate whether it is possible to ascertain homozygous null "human knockouts"; validate loss-of-function experimentally; and do not use constraint alone as a predictor of drug target success.

Critique

The major findings are: (1) while drug targets demonstrate more constraint than the average gene in the human genome, constraint alone is unlikely to be useful in selecting drug targets; (2) the distribution of pLoF variants can be used to predict the number of genes that are likely to harbor homozygous null mutations (i.e., human knockouts) across outbred and special populations; and (3) many predicted LoF variants are likely false positives, and these false positives have a non-random distribution across genes. Based on these findings, the study provides recommendations for the use of human knockouts for drug target evaluation (Box 1).

Overall, I find the topic of great interest, and I believe that the gnomAD dataset provides a very unique resource to answer questions that were previously difficult to address.
They test, for the first time, the value of constraint on drug target success. Their approach is scientifically rigorous, but their conclusions are underwhelming: constraint alone is not a strong predictor of drug target success (see Major Comment #1). They also provide, for the first time, a quantitative assessment of the feasibility of identifying homozygous null mutations for every gene in the genome. I find this result potentially very interesting, and I would like to see the authors provide a more quantitative roadmap of a human knockout project, as well as an online resource that allows investigators to explore feasibility for every gene in the genome (Major Comment #2). Finally, the six use cases highlight the importance of curation to minimize false positive findings. This

finding raises concerns about the conclusions derived on constraint and ascertainment (Major Comment #3).

We thank the reviewer for the deep and thoughtful read of our paper. The reviewer's summary of our work is spot-on, and the framing of this summary has helped us to re-frame our paper around the three main findings. We have re-written the paper mostly from scratch, to now focus on the conclusions that we believe provide the most meaningful guidance for drug discovery efforts. Our responses to specific comments below detail each change.

Major comments

1. Utility of constraint metric. I am struggling to put these results in context. Onone hand, this has been a lingering question in the field which the study addresses in a rigorous manner. On the other hand, the results are less than conclusive – constraint may matter, but it may not. There certainly is value in performing the analysis and reporting the results. But the results are unlikely to be highly impactful for drug discovery.

We thank the reviewer for rightly pointing out that our original manuscript was not very clear about the important takeaway from the constraint analysis. We have reframed the introduction and results to emphasize what we consider to be the core translatable finding here: that even essential genes can make successful drug targets. We believe this is an impactful finding because those of us involved in drug discovery (EVM and SLS) have encountered a widespread attitude, particularly among disease-specific biologists, that "X is not a valid drug target because X is too important". This misperception affects which drug targets are pursued, which grants are funded, and how research is explained to patients. Prior data from knockout mice do support the conclusion that essential genes can be good drug targets, but these data are often discounted due to the potential for discordance between mouse knockout phenotypes and human knockout phenotypes. Thus, providing human data to support this conclusion is important.

In order to better make this case, we have made the following changes: 1) in the introduction, we have cited more of the literature demonstrating that constraint is a meaningful metric of gene essentiality even if it is imperfect (as demonstrated by the curation results later in the paper); 2) in the results, we have reframed the way Figure 1 is introduced, 3) in the results, we have added a brief comparison to mouse knockout data.

In response to the reviewer's suggestion, we also expanded our search for any subset of drug targets where constraint is more informative, adding to Figure 1b a breakdown of drug targets by modality (small molecule, antibody, other) and by indication (oncology, neurology, etc.) — but we still did not find any subset of targets where constraint appears to be more informative.

2. Roadmap for human knockouts. I would like the authors to be more be more quantitative with the number of genes expected to harbor homozygousloss-of-function mutations. There should be a way to integrate data from disease-causing mutations,

***outbred populations, and special populations (e.g., bottleneck, consanguineous) to arrive at a roadmap for a human knockout project. For example, how many genes have human knockouts that lead to severe disease in humans? For the remainder of genes, how many have been identified in extant human knockout databases (e.g., Iceland, PROMISE, ELGH) as harboring homozygous null mutations? Therefore, how many genes have yet to be identified as having homozygous null mutations? Extrapolating from data shown in Figure 4, how many individuals are required to from outbred and special populations to obtain human knockouts for 25%, 50%, and 75% of genes in the human genome? Going further, it should be possible to create an online portal that would allow investigators to estimate the probability of finding human knockouts for every gene in the genome. This could be an interactive portal that would allow users to input numberof genomes sequence across different outbred and special populations*.**

We thank the reviewer for this suggestion, and we agree that this sort of breakdown of human genes is an important gap that we have an opportunity to partially address in this manuscript. We have added such a "roadmap" as Figure 2d-e — we believe that this analysis adds value to the manuscript, and that we have appropriately caveated the assumptions involved in the text.

For the reviewer's benefit, it is also worth explaining a few limitations that cause some details of our new analysis to diverge from the reviewer's vision. First, to the best of our knowledge, there does not exist any database that has accurately and systematically curated all human disease genes to determine which disease associations are attributable to LoF vs. GoF mechanisms (this has been done only for developmental disorder genes in DDG2P and for somatically mutated cancer genes in TCGA). Therefore, while we pulled out a category in Figure 2d for OMIM genes, this include genes for which a GoF disease is known but the effects of LoF are not known, as noted in the text. Second, neither deCODE nor PROMIS offers a public database at this time; public knowledge of homozygous knockouts identified in these cohorts is limited to scientific publications. Thus, while we have included a category for genes with 2-hit genotypes identified in such studies, the gene lists are not all current through 2019, and QC and filtering criteria are not uniform between datasets. Third, creation of an online portal to query such data for each individual gene has been a vision of the MacArthur Lab for some time (a limited prototype is at dblof.broadinstitute.org) but has not yet been funded, and would require a substantial effort beyond the scope of the present study.

3. Curation. Their initial conclusions (constrain, ascertainment) are based on unfiltered pLoF variants. However, in the third section of their study on six neurodegenerative disease genes, they conclude that many predicted LoF variants are false positives. How might these false positives impact their conclusions onconstraint and human knockout ascertainment?

We thank the reviewer for urging us to clarify this point. In fact, the constraint and ascertainment analyses are based on variants that are LOFTEE-filtered but not curated — in the revision we now state this more clearly in Online Methods.

For constraint analyses, it is true that transcript expression-aware annotation and curation would have some impact on the results, but ample evidence, provided mainly by the gnomAD flagship paper (Karczewski et al) indicates that constraint is highly informative despite being imperfect.

We now cite these findings in more detail in the introduction when introducing the constraint concept.

Because false positive LoFs are expected to have higher allele frequencies than true positive LoFs, the impact of curation would be larger for the ascertainment analyses than for the constraint analyses. Therefore, the numbers we present in Figure 2 and in our "roadmap" represent the most optimistic possible picture of the prospects for two-hit LoF ascertainment. We now explicitly make this point in the main text: "Our calculations here likely represent an upper bound on the frequency of two-hit individuals in the population. As a technical matter, the variants included in this analysis are filtered but have not been manually curated or functionally validated, so some will ultimately prove not to be true LoF, and these false positives will have disproportionately contributed to the cumulative LoF allele frequency."

We believe that the ascertainment analysis is valuable in spite of this limitation. As gnomAD and other databases have grown, biologists' expectations have grown in a way that often outstrips reality — some of us have encountered the assumption that if a two-hit genotype for gene X has not yet been identified, then it must not be viable. Our finding that ~1,000 times larger sample sizes are needed to find two-hit individuals for most genes refutes this misperception, sets more realistic expectations for ascertainment, and motivates investment in consanguineous cohorts and in recruitment of LoF heterozygotes for drug target validation. If anything, the fact that true LoF variants may be yet rarer than our analysis assumes only furthers this conclusion.

Minor comments

1. The Abstract provides more background than a summary of the findings in the study, and the Introduction provides a lot of background that is more relevant for a review article. Similarly, the other sections (e.g., constraint, ascertainment, curation)are a bit verbose. Thus, the manuscript could benefit from a more focused writing style throughout.

We have completely re-written the article, reducing its length from ~7,000 words to <3,000, consistent with *Nature* formatting guidelines, and have removed all of the review/perspective/commentary aspects of the text to instead focus exclusively on the original research findings. We believe the new manuscript is much more readable and we thank the reviewer for encouraging us to revise in this manner.

2. The Introduction claims that a "systematic framework for applying human genetic data to the selection of drug targets and to the prediction of drug safety is lacking"(lines 125-127). I believe this is an exaggerated claim, as several nice reviews have been published on this very topic.

The reviewer is correct, and we have removed this claim. In the introduction, we now only say that "Important questions remain, however, regarding strategies for identifying individuals with LoF variants in a gene of interest, interpretation of the frequency — or lack — of such individuals, and whether it is wise to pharmacologically target a gene in which LoF variants are associated with a deleterious phenotype."

3. Lines 198-199 state that the "overall distribution of pLoF obs/exp values for drug targets was similar to that for all genes", but line 253 states "drug targets are on average more depleted for pLoF variation than other genes". These statements appear to contradict each other. Please clarify.

We have revised as follows: "Drug targets were, on average, just slightly more constrained than all genes (mean 44% vs. 52%, P=0.00028), but the two gene sets had a qualitatively similar distribution of scores, ranging from intensely constrained (0% obs/exp) to not at all constrained (≥100% obs/exp; Figure 1a)."

4. Lines 454-456 state "we anticipate that high-throughput direct functional validation of candidate pLoF variants will become the standard for such studies in humans". It would be useful to provide published references to such methodologies.

We have revised this sentence to: "Such curation is essential prior to any recontact efforts, and indeed, establishing methods for high-throughput functional validation48 of LoF variants should be a high priority." Reference 48 here is Jay Shendure's paper on saturation editing of *BRCA1* (PMID: 30209399), which, though focused on missense variants, provides at least some precedent for the development of high-throughput functional validation methodologies. We certainly agree that further development of such methods is needed, and we hope that the rephrasing of this sentence better emphasizes this open need rather than the mere anticipation that it will be accomplished.

Referee #2 (Remarks to the Author):

There's a lot of interest in how human genetics can be exploited to guide drug discovery, in particular for identification and validation of novel targets. The authors have assembled the largest sequencing data set to date to get a systematic and comprehensive view on ultra-rare DNA sequence variation. The specific focus of this paper is on predicted loss-of-function variants that result in human genetic "knockouts" for a gene of interest. The ability to relate such pLoF variants to phenotypes is of great interest: not only for probing the efficacy of gene/protein inhibition with respect to a disease phenotype, but also for assessing potential safety issues for such inhibition. The authors should be congratulated for their intense drive to pull multiple sequencing data sets together in the way that they have, and to create a fantastic open resource that will

allow the community to study a number of very fundamental questions in human genetics.

Although the manuscript is a joy to read (as the authors are careful to walk the reader through some of the intuition or logic of what might be expected in light of evolution and selection), the overall message is one where there's no simple rule to translate the pLoF statistics within a given gene into a meaningful "recommendation" that informs on whether a gene is an efficacious and safe drug target. Despite the fact that there is a statistically significant relative depletion of pLoFs in gene targets of approved drugs (line 212), the overall variation between drug targets (e.g. Table 1) precludes a "simple" rule.
In this sense, the intuition shared by the authors (e.g. lines 191-196; lines 224-225; lines 243-249) is not incredibly helpful to rationalize the presented findings.

We thank the reviewer for this helpful perspective and analysis. While we agree that there is no "simple rule", we have re-written the paper to focus on three key findings: 1) essential/constrained genes can make good drug targets, 2) identification of two-hit LoF individuals is not yet expected for most genes, and 3) curation is important and can identify error modes and/or gene biology. We hope that this re-framing makes the utility of the paper more clear.

Also, the idea that bottlenecked and consanguineous populations may boost power for the characterization of pLoF variants is not so novel anymore, nor is the notion that careful QC filtering and curation is absolutely critical to build such databases. I found the example of the six neurodegenerative genes only mildly useful. The idea that different exons may have different biological functions is obvious, and it follows that the distribution of pLoFs and their relative impact will simply mirror that.

As above, we hope that the re-writing and re-framing of the paper helps bring to the fore those findings that are novel and useful while de-emphasizing the aspects that were already known. Specifically in response to the above points:

1. While the importance of bottlenecked and consanguineous populations is now well- established, we are not aware of any published quantitative estimates of what sample sizes will actually be needed, under various population structure, to identify two-hit individuals for pre- specified genes of interest. The success stories in this area (*PCSK9*, *APOC3* and so on) have led many to expect that other genes of interest will be amenable to human knockout discovery; the finding that we are still three orders of magnitude short for the median gene was surprising to us, and we believe it will be surprising to many readers.

2. By showing that inference of lethality for genes where two-hit individuals are not observed would require yet larger sample sizes, we dispel a common misconception whereby the absence of two-hit individuals in gnomAD is interpreted to mean that this genotype is deleterious or even inviable. Although recent years have seen much discussion and calculation around the sample size required to support *constraint* analyses in genes of different sizes,in

non-coding regions, and in specific exons or protein domains, it is now recognized that constraint mostly reflects selection against heterozygotes (Fuller 2019, PMID: 30962618). To our knowledge, Figure 2 in this paper is the first effort to consider the sample size required to infer non-viability of a homozygous genotype.

3. Although the value of curation may be obvious to people knowledgeable in this area, such as the reviewer, gnomAD serves a diverse audience including clinicians and disease-specific biologists. Our experience has been that for most users it is not at all trivial to look at a gene page and spot which variants are likely false or to recognize what a positional pattern across the gene might imply. We are often contacted by scientists seeking further information about the supposed LoF variants in their gene of interest, and even though LOFTEE filter status (and more recently, exon-specific expression data) are available in the browser, their significance is not widely appreciated. Therefore while perhaps obvious to the reviewer, we believe that the neurodegenerative gene curation exercise will be informative to many readers — and particularly those interested in these specific genes, which are all targets of active preclinical development.

Overall, it feels as though the key messages (e.g. Box 1) could have been better presented in a much more condensed format (perhaps as part of the main paper describing gnomAD). As the authors suggest, "the types of analysis described (…) are only a first step" (lines 557-558).

We have removed Box 1, and we hope that the new paper, at less than half the length of the original, serves the goal of a more condensed format. We thank the reviewer for encouraging us to be more concise.

Referee #3 (Remarks to the Author):

In this paper, Minikel et al. analyze the relevance of metrics of gene essentiality to the selection of drug targets for therapeutic development. A first observation is that the distribution of the pLoF metric for drug targets differs from that of all genes, significantly, but not necessarily meaningfully. In other words, gene essentiality is concluded to not be a strong predictor of whether or not the protein product of a given gene will make a good drug target. A second observation is that LoF mutations are infrequent enough that in outbred populations, "human knockouts" will be vanishingly rare to nonexistent, supporting the case for studying such individuals in consanguineous populations. A third observation is that manual curation of pLoF variants in selected genes associated with neurodegenerative disease reveals challenges related to artifacts, positional distribution, etc. that complicate their interpretation. The collective set of observations are used to provide some guidance for the field in assessing the relevance of pLoF mutations to drug target selection.

We thank the reviewer for this summary and we hope that the rewritten manuscript brings out more clearly the aspects that are novel and applicable to drug discovery efforts.

Overall, this paper is fine in the sense that I don't think that I take strong issue with anything that is said or the analyses done. However, to be frank I found the degree of novelty to be limited. The authors are appropriately conservative in their conclusions, for example not overly interpreting the rather subtle differences in pLoF distributions for drug targets vs. all genes. At the same time, I struggle to identify what is sufficiently new and compelling here that would warrant publication of this paper in Nature. That PCSK9 LoF mutations are well known to be oddly high in frequency -- it was always a bit of a "winner's curse" outlier -- such that knockouts are discoverable, as is the fact that double knockouts for other genes will be very hard to find in outbred populations, and hard-but-not-impossible to find in consanguineous populations. The distribution of pLoF mutations of drug targets is essentially a negative result – a similar result in some sense to the picture that emerges from simply looking at evolutionary conservation (see the overlapping box plot distributions in Fig 2B of https://www.ncbi.nlm.nih.gov/pmc/articles/PMC4826257/). The authors are appropriately conservative in interpreting the moderate difference, which is commendable, but the conclusion (which I agree with) is still that the information (pLOF metrics) is of modestto little value, which somewhat undermines the guidance at the end of the paper (i.e. if knowledge of LoF mutations is not that useful for target selection, then why are we worried about drug companies running afoul of artifacts?).

The reviewer is right that there is already evidence that important/essential/conserved genes can be good drug targets, or may even be enriched among drug targets. As we pointed out in response to reviewer 1, though, this point is not widely known or appreciated among biologists, perhaps in part because existing lines of evidence (e.g. knockout mice) are discounted for not being human data; the same can be said for inter-species conservation data mentioned above (Lv et al 2016, PMID: 26716901).

The fact that some drug targets are highly constrained should not undermine the ascertainment and curation analyses presented later in the paper, because even if the degree of selection against LoF does not predict what is a good drug target, phenotyping of LoF individuals can still be incredibly valuable in validating a therapeutic hypothesis by showing that a reduction in gene dosage brings about the desired phenotypic change. We apologize that our original submission did not do a good enough job of making this distinction. In re-writing the manuscript we have striven to better divide the questions of 1) whether statistical evidence in the absence of phenotype information (e.g. constraint) can help choose drug targets, and 2) how one can identify LoF individuals to phenotype in order to inform drug development.

On both of the above points (finding double-knockouts to gauge safety and using essentiality to pick targets), the authors may be trying to push back against the field overinterpreting the PCSK9 example, but that's really just the field's own fault for being prone to winner's curse rather than a view that's grounded in a credible set of arguments. The arguments about human knockouts and the fact that they won't befound

in outbred populations is correct, but this fact is well predicted by the MAF of LOF mutations and Hardy Weinberg equilibrium.

We agree with the reviewer. While these points may have already been obvious to knowledgeable individuals such as the reviewer, we believe that misconceptions are widespread and that this manuscript provides an important opportunity to dispel them with novel analyses — we are not aware of other published studies we could point to that use cumulative LoF allele frequency to project the sample size that would be needed for ascertainment of two- hit null individuals.

On the last set of observations, regarding challenges in interpreting LoF mutations in databases like ExAC -- again, this is useful, but these kinds of artifacts have been well characterized by the authors and others before, and I'm having trouble finding sufficient novelty here.

As per our response to reviewer 2, we believe that these challenges are appreciated by those knowledgeable in the field but not by a majority of gnomAD users, and that the curation analysis will be of broad interest to many readers

Reviewer Reports on the First Revision:

Referee #1 (Remarks to the Author):

The revised manuscript appropriately focuses on three key areas: (1) "constraint" as a predictor of success for therapeutic targets; (2) ascertainment and availability of human knockouts based on extant databases; and (3) automated vs manual curation for loss-of-function variant annotation, filtering, and interpretation.

First, as in the original submission, the authors conclude that drug targets were slightly more constrained than all genes (mean 44% vs. 52%, P=0.0003), but the two gene sets had a qualitatively similar distribution of scores. Thus, "constraint alone is not adequate to nominate or exclude drug targets".

Second, they estimate the prospects for ascertaining "knockout" individuals in outbred, bottleneck, and consanguineous populations, together with data from Mendelian databases. Of 19,194 human genes, 36% have an observed human knockout; 11% are likely intolerant (and thus knockouts will be observed only in severe or lethal conditions); and 14% do not have sufficient genetic data to infer LoF intolerance. This leaves 7,435 genes (39%) for which one or more pLoFs are observed in gnomAD, but strong LoF intolerance cannot be inferred. The authors conclude, and show nicely in Figures 2d and 2e, that a 1,000-fold increase in sample size (thus >100 million individuals) is required to ascertain human knockouts in outbred populations for these remaining genes, and a 10- to 100-fold increase in sample size (thus >100,000 individuals) to ascertain human knockouts in consanguineous populations.

Third, the authors manually curated gnomAD data and the scientific literature for six genes, demonstrating that many pLoF variants are false positives and that the distribution of pLoF variants can reveal important error modes and insight into disease biology.

Overall, the authors have adequately addressed my concerns. I agree with their response to my Major Comment #1 and appreciate the added analyses shown in Figure 1b. I greatly appreciate the new Figures 2d and 2e, which I find very informative for future prospects of identifying human knockouts. Finally, I find the revised manuscript much more concise and the key messages clearly articulated throughout (e.g., Abstract, Results, Discussion).

Referee #2 (Remarks to the Author):

Essential genes may (or may not) be good drug targets. The obs/exp distribution of pLoF variants in genes is wide - and there's no simple rule that helps predict whether a gene is a good drug target or not. The authors have put together a massive data set and should be commended for sharing it with the community through the gnomAD interface. Insights yielded from single individuals with "knockout" mutations are likely more interesting -- with much increased enthusiasm for detecting such LoF individuals from consanguineous populations (simply due to the inherent relatedness). Essentially this paper provides a framework how to interrogate this important class of variants with increasing investments in sequencing more and more human genomes across populations -- but adds perhaps little to guide the actual selection of good drug targets. That will require (orthogonal) insights into how (and which) genes and pathways underlie key human traits.

Referee #3 (Remarks to the Author):

The paper is largely rewritten and is much clearer both in the writing and message. However, I continue to struggle with the assertion that these are sufficiently novel results to warrant publication at this level.

To go through the three major conclusions:

1. The first conclusion is that (heterozygous) constraint is hardly informative for drug target selection. The nature of the test assumes that drug target selection hasn't been shaped by knowledge of constraint thus far, or of things correlated with constraint, but let's put aside that concern for now. This is a negative result that may surprise some, but if we include the field as a whole, rather than just geneticists, I think that most drug discovery researchers would not be surprised. If we (geneticists) built up expectations about the value of (recent) evolutionary constraint that turn out to be wrong, of course we should show that, but we shouldn't pat ourselves on the backs too much for a subjective expectation that we were responsible in part for setting. Then again, I struggle to think of even whether this expectation was strongly set by anyone. Of course one can point to anecdotal conversations but I can't think of any clear prediction from a prominent geneticist (appearing in the literature rather than conversations) that constraint metrics such as pLOF (obs/exp) that are based on heterozygous mutations in a rapidly expanding population (as opposed to species-level constraint metrics, which have been around for a long time) would strongly correlate with drug target success.

2. The second conclusion is that ascertainment of knockout individuals is extraordinarily inefficient in outbred populations. Again, I agree, but this is very well predicted by population genetics / Hardy-Weinbrerg equilibrium, and already supported by exome/genome data of the past decade. One certainly doesn't need gnomAD to arrive at the same conclusion, and indeed gnomAD is not terribly useful for this question because of the lack of individual-level data (in other words, departures from HWE might make the situation better than it seems, but gnomAD doesn't allow one to evaluate this). Again, I can't think of any prediction in the literature that a 'knockout project' in an outbred population would make any sense. Rather, proposals for knockout projects have been squarely focused on populations with recent or cryptic consanguinaity.

3. The curation of pLOF variants is also fine, and nothing I disagree with, but not very novel. Indeed, early work from this group showed this was the case a number of years ago (i.e. the enrichment for false positives in the most extreme observations). The observations presented here about the nature of these artifacts and the various modes in which they can arise are useful, but at least for me not enough to justify an independent manuscript beyond the primary gnomAD paper in Nature.

Author Rebuttals to First Revision:

Referee #1 (Remarks to the Author):

The revised manuscript appropriately focuses on three key areas: (1) "constraint" as a predictor of success for therapeutic targets; (2) ascertainment and availability of human knockouts based on extant databases; and (3) automated vs manual curation for loss-of- function variant annotation, filtering, and interpretation.

First, as in the original submission, the authors conclude that drug targets were slightly more constrained than all genes (mean 44% vs. 52%, P=0.0003), but the two gene sets had a qualitatively similar distribution of scores. Thus, "constraint alone is not adequate to nominate or exclude drug targets".

Second, they estimate the prospects for ascertaining "knockout" individuals in outbred, bottleneck, and consanguineous populations, together with data from Mendelian databases. Of 19,194 human genes, 36% have an observed human knockout; 11% are likely intolerant (and thus knockouts will be observed only in severe or lethal conditions); and 14% do not have sufficient genetic data to infer LoF intolerance. This leaves 7,435 genes (39%) for which one or more pLoFs are observed in gnomAD, but strong LoF intolerance cannot be inferred. The authors conclude, and show nicely in Figures 2d and 2e, that a 1,000-fold increase in sample size (thus >100 million individuals) is required to ascertain human knockouts in outbred populations for these remaining genes, and a 10- to 100-fold increase in sample size (thus >100,000 individuals) to ascertain human knockouts in consanguineous populations.

Third, the authors manually curated gnomAD data and the scientific literature for six genes, demonstrating that many pLoF variants are false positives and that the distribution of pLoF variants can reveal important error modes and insight into disease biology.

Overall, the authors have adequately addressed my concerns. I agree with their response to my Major Comment #1 and appreciate the added analyses shown in Figure 1b. I greatly appreciate the new Figures 2d and 2e, which I find very informative for future prospects of identifying human knockouts. Finally, I find the revised manuscript much more concise and the key messages clearly articulated throughout (e.g., Abstract, Results, Discussion)

We thank the reviewer for insightful, critical review. We are glad to hear that our revisions have adequately addressed earlier concerns.

Referee #2 (Remarks to the Author):

Essential genes may (or may not) be good drug targets. The obs/exp distribution of pLoF variants in genes is wide - and there's no simple rule that helps predict whether a gene is a good drug target or not. The authors have put together a massive data set and should be commended for sharing it with the community through the gnomAD interface. Insights yielded from single individuals with "knockout" mutations are likely more interesting -- with much increased enthusiasm for detecting such LoF individuals from consanguineous populations (simply due to the inherent relatedness). Essentially this paper provides a framework how to interrogate this important class of variants with increasing investments in sequencing more and more human genomes across populations -- but adds perhaps little to guide the actual selection of good drug targets. That will require (orthogonal) insights into how (and which) genes and pathways underlie key human traits.

We thank the reviewer for the helpful suggestions throughout this process and we agree that there is much work yet to do to improve how genomics is used to select drug targets.

Referee #3 (Remarks to the Author):

The paper is largely rewritten and is much clearer both in the writing and message. However, I continue to struggle with the assertion that these are sufficiently novel results to warrant publication at this level.

To go through the three major conclusions:

1. ***The first conclusion is that (heterozygous) constraint is hardly informative for drug target selection. The nature of the test assumes that drug target selection hasn't been shaped by knowledge of constraint thus far, or of things correlated with constraint, but let's put aside that concern for now. This is a negative result that may surprise some, but if we include the field as a whole, rather than just geneticists, I think that most drug discovery researchers would not be surprised. If we (geneticists) built up expectations about the value of (recent) evolutionary constraint that turn out to be wrong, of course we should show that, but we shouldn't pat ourselves on the backs too much for a subjective expectation that we were responsible in part for setting. Then again, I struggle to think of even whether this expectation was strongly set by anyone. Of course one can point to anecdotal conversations but I can't think of any clear prediction from a prominent geneticist (appearing in the literature rather than conversations) that constraint metrics such as pLOF (obs/exp) that are based on heterozygous mutations in a rapidly expanding population (as opposed to species-level constraint metrics, which have been around for a long time) would strongly correlate with drug target success.***

2. ***The second conclusion is that ascertainment of knockout individuals is extraordinarily inefficient in outbred populations. Again, I agree, but this is very well predicted by population genetics / Hardy-Weinbrerg equilibrium, and already supported by exome/genome data of the past decade. One certainly doesn't need gnomAD to arrive at the same conclusion, and indeed gnomAD is not terribly useful for this question because of the lack of individual-level data (in other words, departures from HWE might make the situation better than it seems, but gnomAD doesn't allow one to evaluate this). Again, I can't think of any prediction in the literature that a 'knockout project' in an outbred population would make any sense. Rather, proposals for knockout projects have been squarely focused on populations with recent or cryptic consanguinaity.***

3. ***The curation of pLOF variants is also fine, and nothing I disagree with, but not very novel. Indeed, early work from this group showed this was the case a number of years ago (i.e. the enrichment for false positives in the most extreme observations). The observations presented here about the nature of these artifacts and the various modes in which they can arise are useful, but at least for me not enough to justify an independent manuscript beyond the primary gnomAD paper in Nature.***

We are grateful to the reviewer for incisive, constructive critiques of our work.
